# Disruptive effects of plasticizers bisphenol A, F, and S on steroidogenesis of adrenocortical cells

**DOI:** 10.3389/fendo.2024.1387133

**Published:** 2024-06-20

**Authors:** Benedikt Pötzl, Lydia Kürzinger, Sabine Kendl, Helga Stopper, Max Kurlbaum, Martin Fassnacht, Ulrich Dischinger

**Affiliations:** ^1^ Division of Endocrinology and Diabetology, Department of Internal Medicine I, University Hospital of Würzburg, Würzburg, Germany; ^2^ Central Laboratory, Core Unit Clinical Mass Spectrometry, University Hospital, University of Würzburg, Würzburg, Germany; ^3^ Institute of Pharmacology and Toxicology, University of Würzburg, Würzburg, Germany

**Keywords:** BPA (bisphenol A), BPF (bisphenol F), BPS (bisphenol S), EDC, endocrine disruptor, adrenal, steroidogenesis, bisphenols

## Abstract

**Introduction:**

Endocrine disrupting chemicals (EDCs) are known to interfere with endocrine homeostasis. Their impact on the adrenal cortex and steroidogenesis has not yet been sufficiently elucidated. This applies in particular to the ubiquitously available bisphenols A (BPA), F (BPF), and S (BPS).

**Methods:**

NCI-H295R adrenocortical cells were exposed to different concentrations (1nM-1mM) of BPA, BPF, BPS, and an equimolar mixture of them (BPmix). After 72 hours, 15 endogenous steroids were measured using LC-MS/MS. Ratios of substrate and product of CYP-regulated steps were calculated to identify most influenced steps of steroidogenesis. mRNA expression of steroidogenic enzymes was determined by real-time PCR.

**Results:**

Cell viability remained unaffected at bisphenol concentrations lower than 250 µM. All tested bisphenols and their combination led to extensive alterations in the quantified steroid levels. The most profound fold changes (FC) in steroid concentrations after exposure to BPA (>10µM) were seen for androstenedione, e.g. a 0.37±0.11-fold decrease at 25µM (p≤0.0001) compared to vehicle-treated controls. For BPF, levels of 17-hydroxyprogesterone were significantly increased by 25µM (FC 2.57±0.49, p≤0.001) and 50µM (FC 2.65±0.61, p≤0.0001). BPS treatment led to a dose-dependent decrease of 11-deoxycorticosterone at >1µM (e.g. FC 0.24±0.14, p≤0.0001 at 10µM). However, when combining all three bisphenols, additive effects were detected: e.g. 11-deoxycortisosterone was decreased at doses >10µM (FC 0.27±0.04, p≤0.0001, at 25µM), whereas 21-deoxycortisol was increased by 2.92±0.20 (p≤0.01) at 10µM, and by 3.21±0.45 (p≤0.001) at 50µM. While every measured androgen (DHEA, DHEAS, androstenedione, testosterone, DHT) was lowered in all experiments, estradiol levels were significantly increased by BPA, BPF, BPS, and BPmix (e.g. FC 3.60±0.54, p≤0.0001 at 100µM BPF). Calculated substrate-product ratios indicated an inhibition of CYP17A1-, and CYP21A2 mediated conversions, whereas CYP11B1 and CYP19A1 showed higher activity in the presence of bisphenols. Based on these findings, most relevant mRNA expression of CYP genes were analysed. mRNA levels of StAR, CYP11B1, and CYP17A1 were significantly increased by BPF, BPS, and BPmix.

**Discussion:**

In cell culture, bisphenols interfere with steroidogenesis at non-cytotoxic levels, leading to compound-specific patterns of significantly altered hormone levels. These results justify and call for additional in-vivo studies to evaluate effects of EDCs on adrenal gland functionality.

## Graphical Abstract

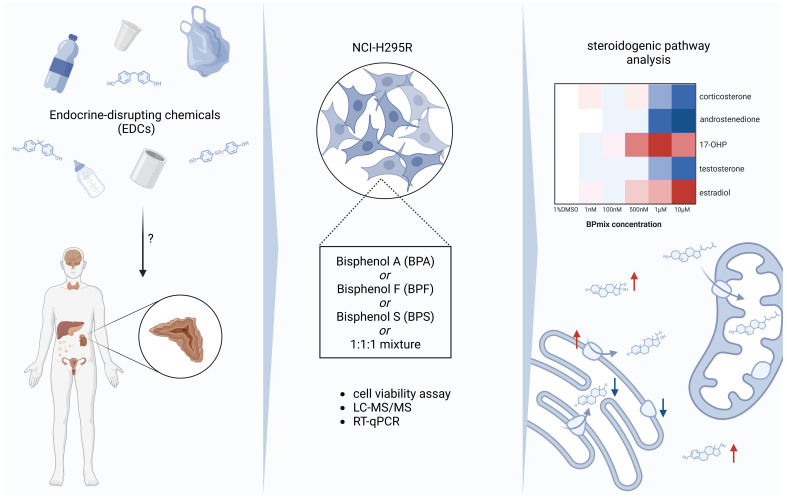

## Introduction

Endocrine disrupting chemicals (EDC) are substances, which interfere with endocrine homeostasis and therefore promote adverse health outcomes (1). Although the evidence for their harmful effects and hazardous potential is growing constantly, there is little regulation on how the disruptive potential of a new substance must be assessed before it is released (2).

Generally, there are numerous chemicals, with which man comes into contact on a daily base, while knowledge is limited about their disrupting potential. EDCs can be found in drinking water and food even years after they have been banned in one region of the world. They can be detected in various human samples like seminal plasma (3), adipose tissue (4), or even amniotic fluid (5). Prominent examples for such EDCs are bisphenol A (BPA) or the structurally similar substances bisphenol F (BPF) and S (BPS), which are nowadays regularly used as substitutes for BPA.

BPA, F and S are aromatic, organic compounds related to diphenylmethane. Used as additive plasticizers in polycarbonate plastics and as primary material for epoxy resins, bisphenols appear in a broad range of applications. They are components of food containers, coatings of food cans, plastic bottles, thermal paper or medical products. In 2021 alone, more than 6 million tons of BPA were produced and distributed worldwide (6). However, not chemically bound to the polymer structure, bisphenols can leach out of the compound, generally promoted by heat, radiation, or weathering processes. Ubiquitously detectable micro- and nanoplastics show high fractions of bisphenols and other EDCs (7). EU-wide biomonitoring was able to detect bisphenols in over 92% of analyzed samples (8). Assuming that the general population is regularly exposed to nanomolar doses, maximum bisphenol levels of 8.48µM (9) or 6.85µM (10) have been measured in occupationally exposed workers. The European Food Safety Authority (EFSA) recently corrected their recommendation for the tolerated intake dose (TDI) for BPA from 4µg/kg body weight/day downwards to 0.2 ng/kg body weight/day following the many indications of risks for public health from the presence of BPA especially in foodstuffs. (7). However, the substitutes BPF and S are still not strictly regulated.

As part of the registration procedure of the OECD (Organization for Economic Co-operation and Development) for substances before approval for distribution, the adrenocortical carcinoma-derived cell line NCI-H295R is used to detect alterations in the secretion by two representative steroids, testosterone and estradiol (11, 12). but further clinically relevant hormones, such as mineralo-, glucocorticoids or adrenal androgens are not taken into account.

Contrary to their effects on the thyroid gland (13, 14), the effects of EDCs on the adrenal gland are largely unknown. Due to its efficient perfusion, lipophilic milieu, and its richness in potential target structures, the adrenal gland appears to be susceptible to the effects of lipophilic EDCs (14). In a cohort study, BPA serum levels seemed to correlate with the diagnosis of non-functional adrenal incidentalomas (15). Additionally, *in vivo* studies revealed BPA’s effect on neurobehavioral endpoints, and an underlying disruption of the hypothalamic-pituitary-adrenal axis, resulting in anxiety-like behavior (16, 17).

The adrenal steroidogenesis underlies a complex regulation by superior hormones, involves multiple cellular organelles, and is dependent on the activity of cytochrome P450 and hydroxysteroid-dehydrogenases. The various, sensitive steps in steroidogenesis can most likely be influenced by EDCs (18, 19), keeping in mind that even minimal changes in steroid levels *in vivo* contribute to disrupted endocrine homeostasis. However, dose-response relationships and toxicological targets of EDCs on adrenal steroidogenesis remain still unclear (20).

Therefore, the present study comprehensively determines the effects of bisphenol A, F and S on the entire adrenal steroidogenesis, including gene expression of key enzymes in the NCI-H295R adrenocortical cell line.

## Materials and Methods

### Chemicals

Bisphenol A (4,4’-(propane-2,2-diyl)diphenol, BPA), bisphenol F (4,4’-methylenediphenol, BPF), and bisphenol S (4,4’-sulfonyldiphenol, BPS) were obtained from Sigma-Aldrich, St. Louis, Missouri, USA. All chemicals were dissolved in dimethyl sulfoxide (DMSO) (Sigma-Aldrich, St. Louis, Missouri, USA) and kept at -20°C. Using 1:1:1 BPA: BPF : BPS, a 100mM stock (BPmix) was obtained, which was used for cell treatment in equimolar concentrations as single bisphenol exposures.

### Cell culture

NCI-H295R cells were grown in Dulbecco’s modified Eagle’s medium/F-12 medium (Gibco, Burlington, Ontario, USA) supplemented with 10% fetal bovine serum (FBS) (Sigma-Aldrich, St. Louis, Missouri, USA) and 1% insulin-transferrin-selenium (ITS) (Sigma-Aldrich, St. Louis, Missouri, USA). Medium was renewed every 3 days. Cells were cultured in 175cm^2^ cell culture flasks (Greiner bio-one, Frickenhausen, Germany) at 37°C with 5% CO2.

Under microscopic control, cells were harvested every week using PBS (Sigma-Aldrich, St. Louis, Missouri, USA) and EDTA-trypsin (Sigma-Aldrich, St. Louis, Missouri, USA). Cell viability was assessed using the Countess II FL (life technologies, Carlsbad, California, USA). If not needed for further experiments, cells were distributed in relation 1:3 into new cell flasks.

For the experiments, passages 16 ± 10 were used. The medium contained 96.05 ± 3.25 ng/l, estradiol, 124.00 ± 77.20 ng/l testosterone, and 105.50 ± 1.50 ng/l progesterone, which was considered in statistical analysis.

### Cell treatment

NCI-H295R cells were seeded in 96-well black plates (Corning Incorporated, Corning, New York, USA) with optically clear flat bottoms at a seeding density of 5000/well in 100µl total volume of complete medium. After 24 h of incubation at 37°C and 5% CO2, cells were exposed for 72 hours to the vehicle (1% DMSO), BPA, BPF, BPS, or the bisphenol mixture (each at 0.001, 0.05, 0.1, 0.25, 0.5, 1, 10, 25, 50, 100, 250, 1000µM). On the day of the experiment, DMSO-dissolved bisphenols were diluted in complete medium to the final concentration assuring the DMSO concentration was 1%. A total volume of 100µL was added to each well.

At the end of the incubation period, 150µL supernatant of eight parallel treated wells were collected, centrifuged at 800 rpm (23°C), and stored at -20°C until steroid quantification.

### Cell viability assay

Cell viability was measured using the CellTiter-Glo^®^ Luminescent assay (Promega, Madison, Wisconsin, USA) following the manufacturer’s protocol. After treatment, the cells were incubated with CellTiter-Glo^®^ Reagent for 10 min. After incubation, the luminescence of each well was determined using 1420 multilabel counter Victor^3^ (Perkin Elmer, Waltham, Massachusetts, USA).

Each experiment was performed in three independent experiments in 8-plicates, and the results are expressed as fold changes to vehicle-treated cells.

### Steroid hormone analysis

Liquid chromatography tandem mass spectrometry (LC-MS/MS) was performed using a Sciex 6500+ QTRAP (SCIEX, Framingham, USA) MS-system linked with an Agilent 1290 HPLC-system (G4226A autosampler, infinityBinPump, G1316C column-oven, G1330B thermostat; Santa Clara, USA). The quantification of 15 steroid hormones (aldosterone, androstenedione, corticosterone, cortisol, cortisone, dehydroepiandrostendion (DHEA), dehydroepiandrostendion-sulfate (DHEAS), 11-deoxycorticosterone, 11-deoxycortisol, 21-deoxycortisol, Dihydrotestosterone (DHT), estradiol, 17-hydroxyprogesterone (17-OHP), progesterone, and testosterone) via MRM was performed with the MassChrom-Steroids in Serum/Plasma^®^ IVDR conform kit (Chromsystems^®^, Graüfelfing) as described in detail elsewhere (21). After off-line solid phase extraction of 500 μL cell supernatant, 15 μL were used for analysis. Concentrations were calculated with Analyst Software (1.6.3) via six-point calibration and 1/× weighting. Correctness of measurements was controlled by commercial quality controls and periodic participation in ring trails.

### RNA Extraction and reverse transcription

For RNA extraction, NCI-H295R cells were seeded in 12 well plates (Corning Incorporated, Corning, New York, USA) at a density of 1x10^6 cells/well and exposed to 1% dimethyl sulfoxide (DMSO), BPA, BPF BPS, or the bisphenol mixture (each at 0.1, 1, 10, 25, 50µM). After 72 hours, cells were harvested using PBS and EDTA-trypsin. Cell pellets were centrifuged and washed with PBS. RNA was extracted using the Maxwell^®^ RSC simply RNA Tissue kit (Promega, Madison, Wisconsin, USA) following manufacturer’s protocol, and stored at -80°C for later reverse transcription.

A total of 1µg RNA per sample was reverse-transcribed using the QuantiTect^®^ Reverse Transcription kit (Qiagen, Venlo, Netherlands) in a final volume of 40µL. cDNA was diluted by 1:10 in nuclease-free water and stored at -20°C for further analysis.

### Quantitative real-time PCR

The RT-PCR reactions were performed using C1000 Thermal Cycler (biorad, Hercules, California, USA) with a reaction mixture comprised of 12.5µg water, 8µg TaqMan Gene Expression Master Mix (Thermo Fischer Scientific, Waltham, Massachusetts, USA), 8µg for each primer (Thermo Fischer Scientific, Waltham, Massachusetts, USA, see **Supplementary Table 1**), 2µg for each cDNA/well. Beta-Actin (ACTB) was used as a housekeeping gene.

mRNA levels were measured with quantitative real-time RT-PCR by the comparative Ct (^2-ΔΔ^Ct) method using qRAT expression analysis (22).

Each experiment was repeated three times in duplicates, and the results are demonstrated as fold changes in comparison to vehicle-treated cells.

### Statistical analysis

Statistical analysis was performed using GraphPad Prism Software (version 9.01 for Windows, GraphPad Software, Inc.). Data from cell viability, steroid quantification in response to bisphenol concentrations, and gene expression were analyzed using one-way ANOVA with Dunnett’s *post-hoc* comparisons. Differences between vehicle- and BPA-/BPF-/BPS- or mixture-treated cells were analyzed using one-way-ANOVA and Dunnett*s *post-hoc* multiple comparisons. A p ≤ 0.05 was considered statistically significant.

**Figure 1:**
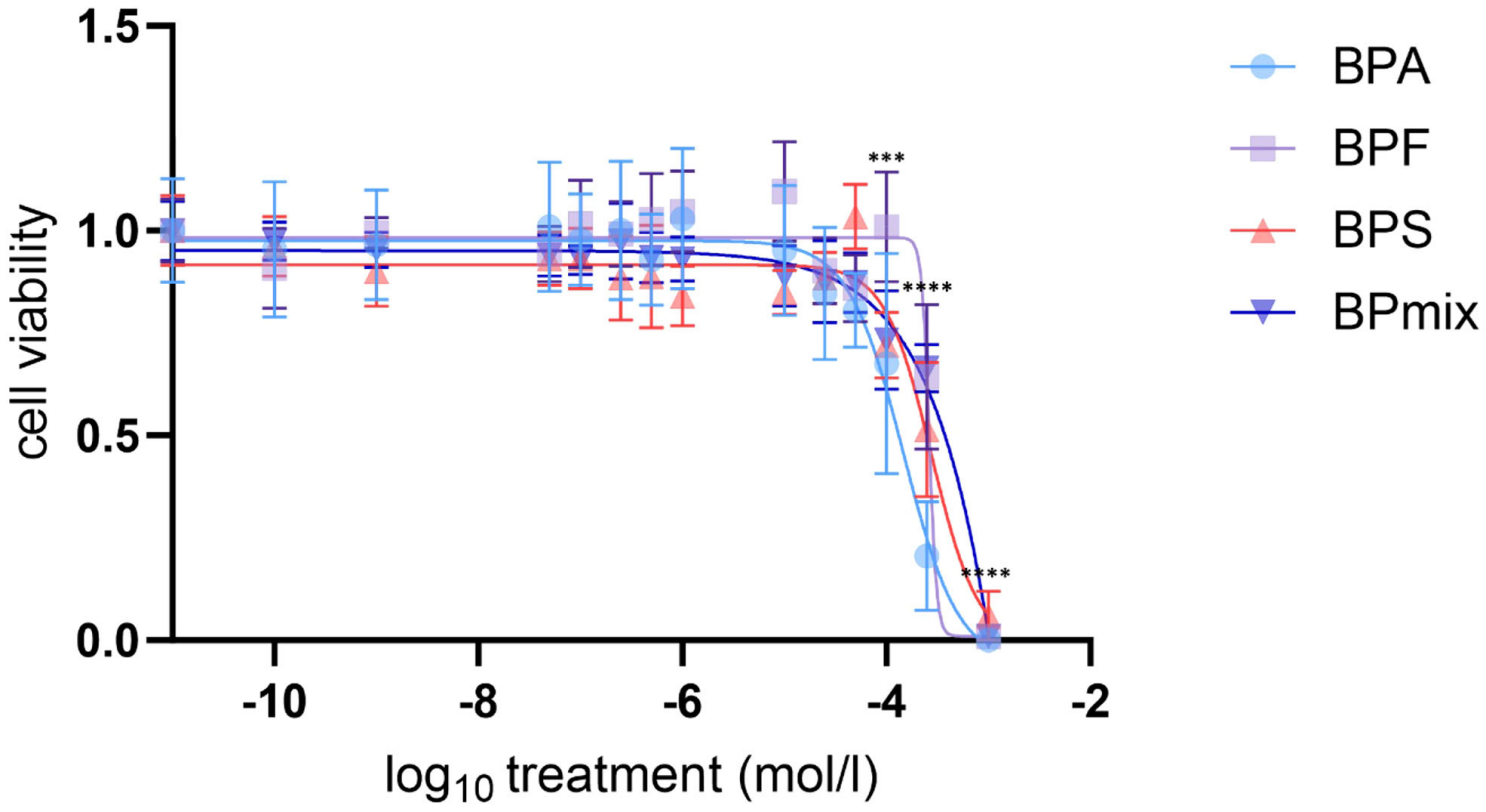
Cell viability of NCI-H295R after 72h treatment with BPA, F and S or mixture. Treatment concentration related (log_10_) mean values compared to vehicle-treated control ± SD, n=3. *** p≤0.001, **** p≤0.0001.

**Figure 2:**
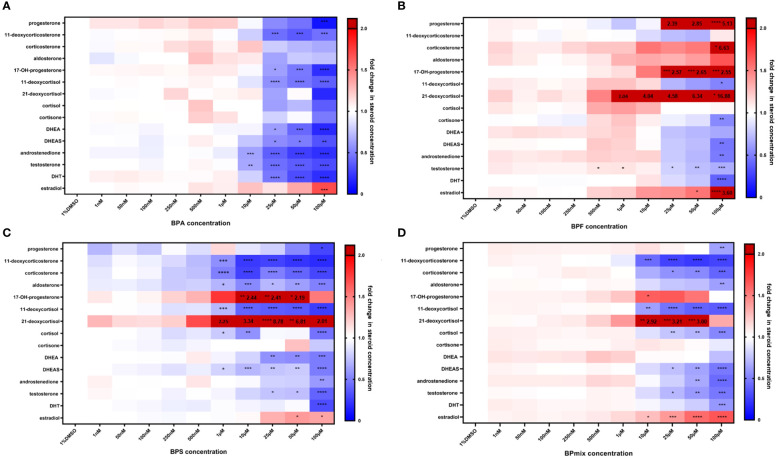
Steroid concentrations after treatment of NCI H295 cells with BPA **(A)**, BPF **(B)**, BPS **(C)**, BPmix **(D)** or vehicle control (1% DMSO) for 72 hours. Blue marks a decrease, whereas red indicates an increase in steroid concentration. Values exceeding the given scale are illustrated in dark red with respective values. n=3. * p≤0.05, ** p≤0.01, *** p≤0.001, **** p≤0.0001 compared to vehicle-treated control.

**Figure 3:**
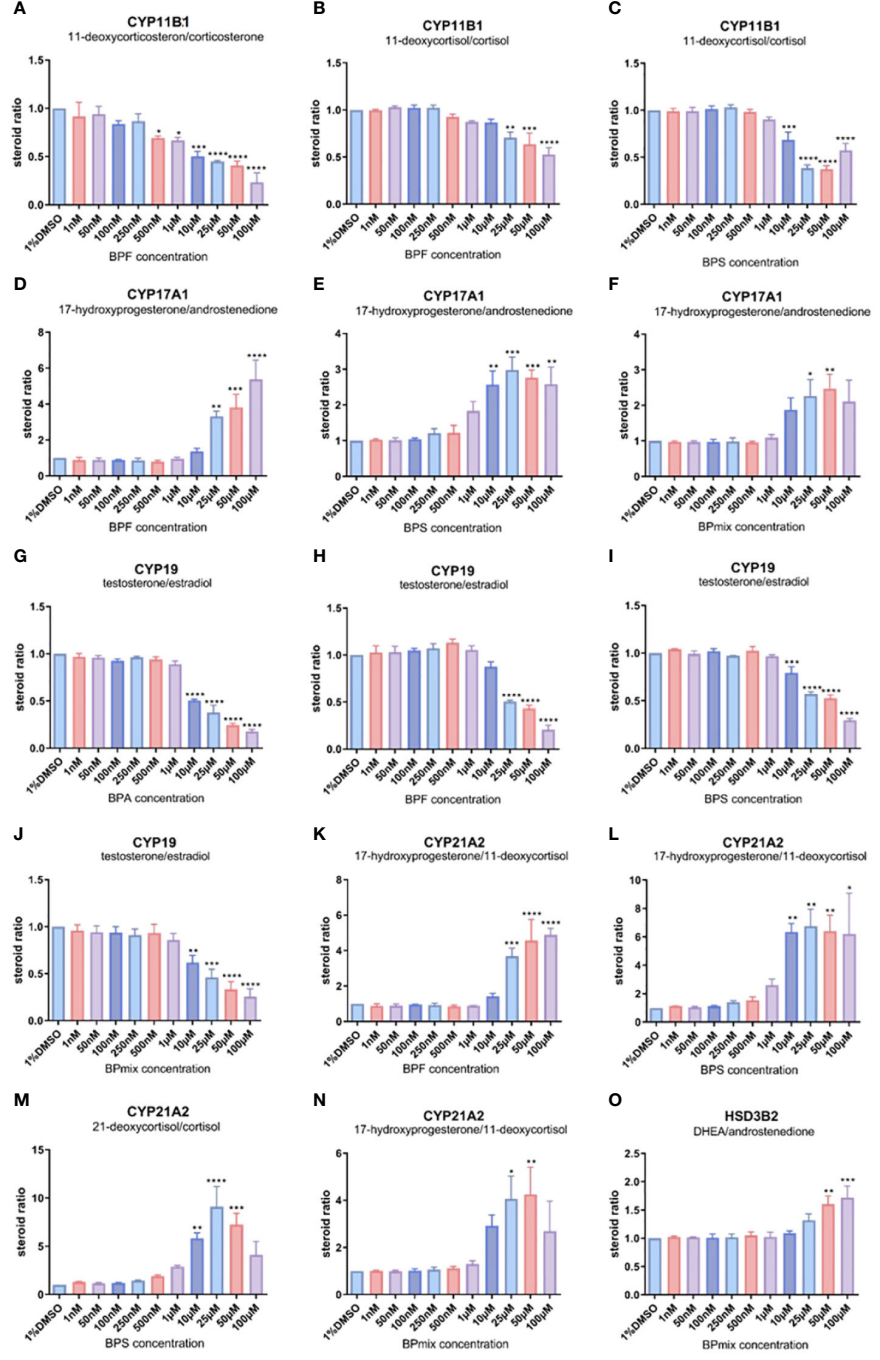
Ratios of substrate/product of different steroid metabolism related CYP-enzymes compared to vehicle-treated control. Most significant changes were seen for CYP11B1 **(A–C)**, CYP17A1 **(D–F)**, CYP19 **(G–J)**, CYP21A2 **(K–N)**, and HSD3B2 **(O)**. Remaining steroid ratios are available in **Supplementary Material**. Values >1 indicate a reduced conversion of the substrate, while values<1 suggest an enhanced activity of the certain steroidogenic step. Fold change ± SD. n=3. * p≤0.05, ** p≤0.01, *** p≤0.001, **** p≤0.0001.

**Figure 4:**
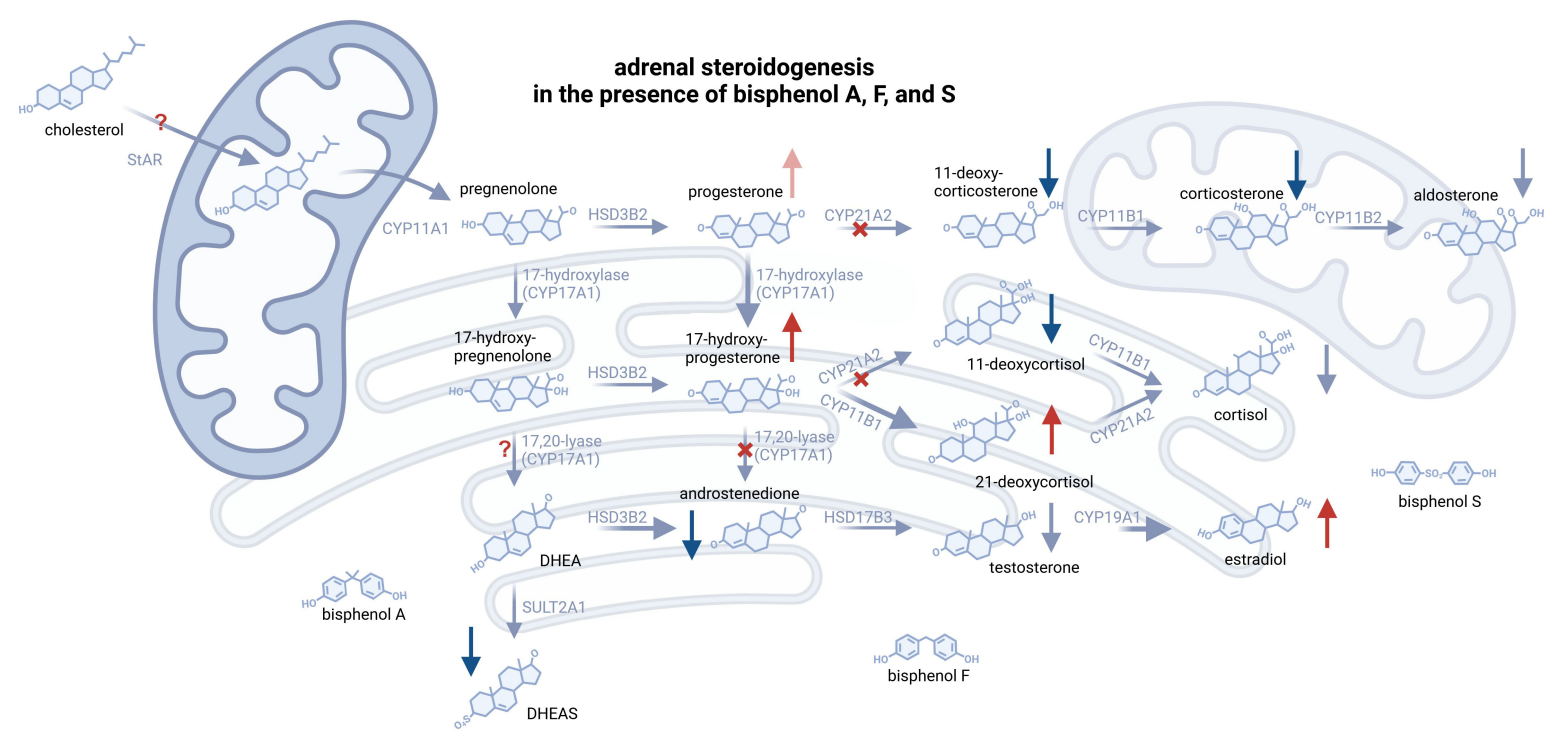
Illustration of substantial alterations on adrenal steroidogenesis, induced by an equimolar mixture of BPA, BPF, and BPS at doses >10µM. Enhanced conversions are displayed as bolder arrows, whereas inhibited steps are marked with a red X. Red and blue arrows indicate increased or decreased concentrations of the respective steroid. Some essential steroidogenic steps could not have been elucidated and are therefore marked with “?”. Peripheral steroids, such as cortisone and DHT, are not shown. Created with biorender.com.

**Figures 1**–**3** were designed in GraphPad Prism. The graphical abstract and **Figure 4** were created with biorender.com.

## Results

### Cell viability

There was no significant difference in cell viability between untreated and vehicle-treated cells. Cell viability remained unaffected at lower bisphenol concentrations but decreased to 68% (p ≤ 0.001) by BPA, to 72% (p ≤ 0.001) by BPS and to 73% (p ≤ 0.001) by BPmix. For each substance, as well as for the mixture viability decreased to<66% (p ≤ 0.0001) at 250µM and to<6% (p ≤ 0.0001) compared to vehicle-treated control at 1mM. Calculated median lethal doses (LC_50_) were 146.6µM for BPA, 263.6µM for BPF, 278.0µM for BPS, and 272.8µM for BPmix (**Figure 1**).

### Steroid secretion

Concentrations, which caused pronounced reduction in cell viability, led to significant decrease in all steroid values as well. Therefore, the results obtained at these concentrations (250 µM and 1mM) are excluded from further analyses. Fold changes in steroid levels in the presence of 1nM to 100µM BPA, BPF, BPS, or bisphenol mixture (BPmix) are presented in **Table 1** and **Figure 2**.

**Table 1:** Fold changes of measured steroid concentrations after 72h of treatment with BPA, BPF, BPS, or BPmix.

BPA	1nM	50nM	100nM	250nM	500nM	1µM	10µM	25µM	50µM	100µM	250µM	1mM
progesterone	1.16 ± 0.34	1.15 ± 0.20	1.23 ± 0.34	1.17 ± 0.53	1.28 ± 0.28	1.28 ± 0.28	0.95 ± 0.13	0.54 ± 0.14	0.45 ± 0.40	**0.10 ± 0.10**	**0.23 ± 0.06**	**0.08 ± 0.10**
11-deoxycorticosterone	0.96 ± 0.16	1.03 ± 0.06	1.04 ± 0.14	1.05 ± 0.20	1.12 ± 0.17	0.97 ± 0.05	0.79 ± 0.07	**0.44 ± 0.10**	**0.36 ± 0.14**	**0.45 ± 0.22**	0.87 ± 0.13	1.09 ± 0.21
corticosterone	0.95 ± 0.09	1.06 ± 0.09	1.05 ± 0.06	1.20 ± 0.06	1.26 ± 0.27	1.08 ± 0.12	1.31 ± 0.33	0.78 ± 0.11	0.71 ± 0.47	0.65 ± 0.31	1.52 ± 0.41	2.34 ± 0.70
aldosterone	0.88 ± 0.03	0.95 ± 0.30	0.96 ± 0.06	0.99 ± 0.20	1.34 ± 0.53	1.17 ± 0.08	1.13 ± 0.38	0.63 ± 0.23	0.78 ± 0.82	0.74 ± 0.13	0.86 ± 0.15	0.57 ± 0.17
17-OH-progesterone	1.05 ± 0.04	1.06 ± 0.03	1.08 ± 0.05	1.05 ± 0.12	1.12 ± 0.08	1.12 ± 0.12	0.96 ± 0.06	**0.52 ± 0.21**	**0.47 ± 0.36**	**0.19 ± 0.05**	**0.13 ± 0.01**	**0.07 ± 0.02**
11-deoxycortisol	0.99 ± 0.04	1.04 ± 0.03	1.00 ± 0.02	1.05 ± 0.06	1.09 ± 0.20	1.04 ± 0.13	0.87 ± 0.02	**0.62 ± 0.08**	**0.46 ± 0.14**	**0.38 ± 0.08**	**0.39 ± 0.04**	**0.43 ± 0.04**
21-deoxycortisol	0.95 ± 0.01	0.97 ± 0.14	1.02 ± 0.02	1.22 ± 0.09	1.04 ± 0.05	1.07 ± 0.21	1.12 ± 0.09	0.63 ± 0.31	1.04 ± 0.14	0.17 ± 0.01	**0.10 ± 0.01**	**0.13 ± 0.01**
cortisol	0.98 ± 0.03	1.02 ± 0.08	0.99 ± 0.08	1.04 ± 0.16	1.31 ± 0.61	1.01 ± 0.22	1.01 ± 0.13	0.62 ± 0.17	0.72 ± 0.70	0.37 ± 0.10	**0.29 ± 0.06**	**0.31 ± 0.06**
cortisone	1.05 ± 0.10	1.06 ± 0.11	1.02 ± 0.12	0.99 ± 0.15	1.27 ± 0.48	1.17 ± 0.23	0.99 ± 0.10	0.81 ± 0.13	0.95 ± 0.58	0.48 ± 0.11	**0.19 ± 0.03**	**0.13 ± 0.01**
DHEA	0.96 ± 0.20	0.97 ± 0.16	0.94 ± 0.11	1.02 ± 0.29	1.00 ± 0.21	0.94 ± 0.21	0.93 ± 0.09	**0.61 ± 0.14**	**0.41 ± 0.27**	**0.17 ± 0.10**	0.49 ± 0.23	0.54 ± 0.40
DHEAS	0.98 ± 0.14	0.97 ± 0.08	0.91 ± 0.06	0.97 ± 0.10	1.10 ± 0.39	1.03 ± 0.24	0.76 ± 0.11	**0.76 ± 0.18**	**0.41 ± 0.38**	**0.33 ± 0.15**	0.26 ± 0.12	0.23 ± 0.13
androstenedione	0.94 ± 0.16	0.94 ± 0.13	0.92 ± 0.10	0.99 ± 0.12	0.97 ± 0.16	0.91 ± 0.12	**0.63 ± 0.05**	**0.37 ± 0.11**	**0.25 ± 0.10**	**0.20 ± 0.03**	**0.31 ± 0.06**	**0.31 ± 0.08**
testosterone	0.98 ± 0.09	1.00 ± 0.07	0.95 ± 0.05	0.99 ± 0.09	1.07 ± 0.27	0.97 ± 0.15	**0.65 ± 0.05**	**0.44 ± 0.14**	**0.37 ± 0.15**	**0.30 ± 0.03**	**0.23 ± 0.02**	**0.12 ± 0.01**
DHT	1.09 ± 0.03	1.12 ± 0.07	1.09 ± 0.06	1.04 ± 0.10	1.05 ± 0.11	1.10 ± 0.05	0.81 ± 0.04	**0.47 ± 0.27**	**0.31 ± 0.10**	**0.19 ± 0.10**	**0.13 ± 0.01**	**0.06 ± 0.01**
estradiol	1.01 ± 0.03	1.05 ± 0.04	1.03 ± 0.04	1.03 ± 0.08	1.16 ± 0.31	1.11 ± 0.18	1.33 ± 0.15	1.16 ± 0.03	1.46 ± 0.40	**1.82 ± 0.47**	1.41 ± 0.21	1.00 ± 0.10
BPF	1nM	50nM	100nM	250nM	500nM	1µM	10µM	25µM	50µM	100µM	250µM	1mM
progesterone	1.10 ± 0.24	1.08 ± 0.31	0.98 ± 0.28	1.04 ± 0.11	0.92 ± 0.36	0.76 ± 0.24	0.94 ± 0.09	2.39 ± 0.88	2.85 ± 1.00	**5.13 ± 2.10**	**8.19 ± 2.01**	0.54 ± 0.15
11-deoxycorticosterone	1.09 ± 0.20	1.04 ± 0.15	0.95 ± 0.11	1.06 ± 0.08	1.02 ± 0.19	0.97 ± 0.17	0.84 ± 0.06	0.73 ± 0.02	0.69 ± 0.08	1.13 ± 1.05	1.05 ± 0.23	1.03 ± 0.31
corticosterone	1.24 ± 0.37	1.13 ± 0.29	1.13 ± 0.07	1.24 ± 0.18	1.32 ± 0.04	1.32 ± 0.24	1.71 ± 0.35	1.62 ± 0.05	1.70 ± 0.15	**6.63 ± 2.75**	1.49 ± 0.48	1.41 ± 0.55
aldosterone	1.19 ± 0.22	1.11 ± 0.16	1.04 ± 0.08	1.08 ± 0.06	1.15 ± 0.08	1.41 ± 0.14	1.46 ± 0.32	1.42 ± 0.24	1.52 ± 0.49	1.88 ± 1.55	0.53 ± 0.01	0.51 ± 0.04
17-OH-progesterone	0.98 ± 0.02	0.98 ± 0.07	1.01 ± 0.08	1.00 ± 0.06	1.11 ± 0.21	1.19 ± 0.26	1.69 ± 0.44	**2.57 ± 0.49**	**2.65 ± 0.61**	**2.55 ± 0.79**	1.30 ± 0.22	**0.08 ± 0.01**
11-deoxycortisol	1.14 ± 0.25	1.11 ± 0.17	1.05 ± 0.12	1.09 ± 0.16	1.34 ± 0.47	1.33 ± 0.30	1.21 ± 0.29	0.70 ± 0.01	0.61 ± 0.11	**0.51 ± 0.10**	**0.42 ± 0.01**	**0.39 ± 0.03**
21-deoxycortisol	1.26 ± 0.29	1.09 ± 0.23	1.20 ± 0.20	1.11 ± 0.19	1.65 ± 0.70	2.04 ± 0.49	4.04 ± 1.56	4.58 ± 2.04	6.33 ± 4.75	**16.88 ± 4.01**	0.97 ± 0.21	0.20 ± 0.01
cortisol	1.14 ± 0.26	1.07 ± 0.15	1.03 ± 0.06	1.06 ± 0.12	1.46 ± 0.56	1.52 ± 0.38	1.41 ± 0.42	1.00 ± 0.14	0.99 ± 0.18	1.06 ± 0.50	0.41 ± 0.04	**0.26 ± 0.03**
cortisone	0.97 ± 0.05	0.99 ± 0.04	0.94 ± 0.04	0.92 ± 0.06	1.20 ± 0.29	1.32 ± 0.27	1.11 ± 0.22	0.92 ± 0.03	0.87 ± 0.06	**0.54 ± 0.08**	**0.37 ± 0.01**	**0.12 ± 0.02**
DHEA	1.15 ± 0.45	1.20 ± 0.28	1.18 ± 0.24	1.15 ± 0.37	1.22 ± 0.14	1.28 ± 0.19	1.03 ± 0.08	0.75 ± 0.02	0.71 ± 0.07	0.65 ± 0.13	0.36 ± 0.11	0.96 ± 0.55
DHEAS	1.06 ± 0.24	1.05 ± 0.12	1.02 ± 0.09	1.01 ± 0.17	1.30 ± 0.27	1.24 ± 0.08	1.04 ± 0.34	0.77 ± 0.08	0.73 ± 0.13	**0.46 ± 0.34**	**0.38 ± 0.21**	0.62 ± 0.01
androstenedione	1.16 ± 0.32	1.13 ± 0.19	1.15 ± 0.16	1.19 ± 0.24	1.27 ± 0.24	1.24 ± 0.17	1.25 ± 0.30	0.77 ± 0.03	0.70 ± 0.06	**0.48 ± 0.08**	**0.40 ± 0.09**	**0.37 ± 0.11**
testosterone	1.15 ± 0.24	1.09 ± 0.15	1.08 ± 0.12	1.09 ± 0.16	1.15 ± 0.07	1.16 ± 0.04	1.10 ± 0.10	**0.81 ± 0.14**	**0.73 ± 0.05**	**0.67 ± 0.20**	0.52 ± 0.08	**0.19 ± 0.04**
DHT	0.99 ± 0.04	1.00 ± 0.03	0.97 ± 0.04	0.97 ± 0.02	0.99 ± 0.08	1.01 ± 0.03	1.17 ± 0.23	0.84 ± 0.04	0.77 ± 0.09	**0.39 ± 0.20**	**0.22 ± 0.05**	**0.07 ± 0.01**
estradiol	1.10 ± 0.09	1.06 ± 0.06	1.03 ± 0.07	1.01 ± 0.07	1.24 ± 0.35	1.29 ± 0.24	1.56 ± 0.44	1.59 ± 0.21	**1.72 ± 0.36**	**3.60 ± 0.58**	**2.64 ± 0.29**	1.06 ± 0.27
BPS	1nM	50nM	100nM	250nM	500nM	1µM	10µM	25µM	50µM	100µM	250µM	1mM
progesterone	0.71 ± 0.31	0.85 ± 0.47	0.77 ± 0.34	0.96 ± 0.55	0.85 ± 0.48	1.16 ± 0.75	0.89 ± 0.52	0.80 ± 0.06	0.53 ± 0.09	**0.28 ± 0.09**	0.09 ± 0.05	0.08 ± 0.06
11-deoxycorticosterone	0.85 ± 0.04	0.95 ± 0.09	0.93 ± 0.09	0.84 ± 18	0.72 ± 0.16	**0.56 ± 0.19**	**0.24 ± 0.14**	**0.27 ± 0.18**	**0.22 ± 0.15**	**0.18 ± 0.09**	**0.45 ± 0.08**	0.84 ± 0.17
corticosterone	0.89 ± 0.14	0.99 ± 0.20	0.94 ± 0.15	0.77 ± 0.12	0.74 ± 0.15	**0.50 ± 0.11**	**0.26 ± 0.07**	**0.50 ± 0.14**	**0.40 ± 0.07**	**0.28 ± 0.07**	**0.50 ± 0.08**	0.84 ± 0.23
aldosterone	0.91 ± 0.15	0.93 ± 0.13	0.88 ± 0.12	0.78 ± 0.13	0.80 ± 0.20	**0.70 ± 0.27**	**0.54 ± 0.32**	**0.71 ± 0.03**	**0.61 ± 0.03**	**0.50 ± 0.17**	0.46 ± 0.06	0.45 ± 0.10
17-OH-progesterone	1.10 ± 0.04	1.02 ± 0.06	1.06 ± 0.04	1.19 ± 0.17	1.31 ± 0.19	1.78 ± 0.24	**2.44 ± 0.61**	**2.41 ± 0.47**	**2.19 ± 0.20**	1.52 ± 1.14	0.68 ± 0.12	0.10 ± 0.01
11-deoxycortisol	0.98 ± 0.05	0.97 ± 0.07	0.96 ± 0.11	0.86 ± 0.14	0.87 ± 0.16	**0.71 ± 0.16**	**0.39 ± 0.13**	**0.37 ± 0.04**	**0.35 ± 0.06**	**0.25 ± 0.03**	**0.28 ± 0.02**	**0.30 ± 0.02**
21-deoxycortisol	1.29 ± 0.15	1.16 ± 0.26	1.14 ± 0.16	1.20 ± 0.25	1.70 ± 0.35	2.25 ± 0.38	3.34 ± 1.01	**8.78 ± 1.00**	**6.81 ± 0.50**	2.01 ± 0.40	0.52 ± 0.07	0.23 ± 0.07
cortisol	0.99 ± 0.10	0.99 ± 0.14	0.95 ± 0.13	0.84 ± 0.16	0.89 ± 0.16	**0.78 ± 0.13**	**0.56 ± 0.09**	0.95 ± 0.06	0.94 ± 0.01	**0.45 ± 0.10**	**0.33 ± 0.04**	**0.22 ± 0.05**
cortisone	1.00 ± 0.04	0.96 ± 0.06	0.94 ± 0.07	0.91 ± 0.09	0.93 ± 0.04	1.01 ± 0.08	1.02 ± 0.14	0.99 ± 0.05	1.26 ± 0.02	0.77 ± 0.28	**0.34 ± 0.02**	**0.13 ± 0.03**
DHEA	1.01 ± 0.05	0.99 ± 0.12	0.98 ± 0.04	0.97 ± 0.12	1.09 ± 0.28	0.93 ± 0.20	0.82 ± 0.15	**0.57 ± 0.03**	**0.55 ± 0.04**	**0.48 ± 0.31**	0.49 ± 0.18	1.36 ± 0.77
DHEAS	1.01 ± 0.04	0.94 ± 0.03	0.93 ± 0.03	0.87 ± 0.04	0.91 ± 0.08	**0.81 ± 0.14**	**0.70 ± 0.14**	**0.76 ± 0.02**	**0.78 ± 0.04**	**0.48 ± 0.01**	**0.38 ± 0.06**	**0.30 ± 0.08**
androstenedione	1.07 ± 0.03	1.01 ± 0.08	1.02 ± 0.08	0.98 ± 0.09	1.12 ± 0.24	0.99 ± 0.11	0.96 ± 0.15	0.81 ± 0.03	0.79 ± 0.04	**0.59 ± 0.42**	**0.51 ± 0.17**	**0.33 ± 0.10**
testosterone	1.05 ± 0.04	0.97 ± 0.05	0.98 ± 0.10	0.93 ± 0.11	1.02 ± 0.18	0.94 ± 0.13	0.82 ± 0.19	**0.75 ± 0.02**	**0.73 ± 0.07**	**0.39 ± 0.18**	**0.22 ± 0.03**	**0.10 ± 0.02**
DHT	0.98 ± 0.01	0.96 ± 0.03	0.95 ± 0.09	0.92 ± 0.06	0.91 ± 0.07	0.97 ± 0.12	0.93 ± 0.11	0.91 ± 0.05	0.96 ± 0.03	**0.31 ± 0.06**	**0.16 ± 0.05**	**0.07 ± 0.01**
estradiol	1.01 ± 0.04	0.99 ± 0.10	0.96 ± 0.09	0.95 ± 0.11	0.99 ± 0.10	0.97 ± 0.11	1.04 ± 0.10	1.32 ± 0.08	**1.40 ± 0.05**	**1.36 ± 0.47**	1.08 ± 0.14	0.70 ± 0.15
BPmix	1nM	50nM	100nM	250nM	500nM	1µM	10µM	25µM	50µM	100µM
progesterone	1.12 ± 0.16	1.10 ± 0.18	1.09 ± 0.25	1.09 ± 0.27	1.08 ± 0.14	1.10 ± 0.24	1.18 ± 0.51	1.09 ± 0.59	1.00 ± 0.53	**0.63 ± 0.59**
11-deoxycorticosterone	1.09 ± 0.18	1.06 ± 0.22	1.07 ± 0.20	1.01 ± 0.22	0.97 ± 0.24	0.91 ± 0.29	**0.42 ± 0.05**	**0.27 ± 0.04**	**0.23 ± 0.02**	**0.27 ± 0.08**
corticosterone	1.03 ± 0.27	1.04 ± 0.30	1.06 ± 0.24	1.11 ± 0.26	1.10 ± 0.43	1.03 ± 0.53	0.67 ± 0.37	**0.57 ± 0.32**	**0.54 ± 0.30**	**0.39 ± 0.22**
aldosterone	1.05 ± 0.25	1.05 ± 0.22	1.05 ± 0.23	1.00 ± 0.25	1.06 ± 0.29	1.05 ± 0.35	0.84 ± 0.24	0.76 ± 0.16	0.74 ± 0.15	**0.62 ± 0.06**
17-OH-progesterone	1.09 ± 0.19	1.08 ± 0.22	1.09 ± 0.19	1.10 ± 0.25	1.19 ± 0.38	1.31 ± 0.43	**1.83 ± 0.79**	1.86 ± 0.96	1.71 ± 0.72	1.00 ± 0.88
11-deoxycortisol	1.08 ± 0.14	1.09 ± 0.17	1.07 ± 0.15	1.04 ± 0.21	1.06 ± 0.28	1.01 ± 0.30	**0.61 ± 0.12**	**0.45 ± 0.09**	**0.39 ± 0.06**	**0.36 ± 0.02**
21-deoxycortisol	1.06 ± 0.31	1.04 ± 0.29	1.10 ± 0.27	1.13 ± 0.34	1.26 ± 0.59	1.54 ± 0.77	**2.92 ± 0.20**	**3.21 ± 0.45**	**2.92 ± 0.32**	1.45 ± 1.79
cortisol	1.11 ± 0.20	1.10 ± 0.19	1.09 ± 0.15	1.06 ± 0.22	1.09 ± 0.31	1.09 ± 0.35	0.86 ± 0.25	**0.76 ± 0.19**	**0.70 ± 0.20**	**0.53 ± 0.19**
cortisone	1.11 ± 0.19	1.09 ± 0.21	1.05 ± 0.15	1.05 ± 0.17	1.05 ± 0.25	1.10 ± 0.30	1.15 ± 0.45	1.11 ± 0.44	1.05 ± 0.48	0.90 ± 0.64
DHEA	1.15 ± 0.20	1.14 ± 0.18	1.14 ± 0.31	1.16 ± 0.34	1.31 ± 0.52	1.26 ± 0.52	1.04 ± 0.05	1.04 ± 0.10	1.01 ± 0.16	0.73 ± 0.38
DHEAS	1.09 ± 0.21	1.06 ± 0.20	1.02 ± 0.20	1.01 ± 0.22	1.06 ± 0.34	1.04 ± 0.32	0.84 ± 0.11	**0.74 ± 0.18**	**0.64 ± 0.23**	**0.47 ± 0.29**
androstenedione	1.12 ± 0.16	1.11 ± 0.18	1.12 ± 0.18	1.13 ± 0.23	1.23 ± 0.42	1.20 ± 0.40	0.96 ± 0.27	0.80 ± 0.24	**0.65 ± 0.22**	**0.42 ± 0.18**
testosterone	1.05 ± 0.25	1.04 ± 0.26	1.02 ± 0.21	1.00 ± 0.23	1.08 ± 0.42	1.06 ± 0.40	0.85 ± 0.27	**0.70 ± 0.24**	**0.59 ± 0.27**	**0.47 ± 0.23**
DHT	1.12 ± 0.19	1.09 ± 0.19	1.06 ± 0.16	1.07 ± 0.19	1.09 ± 0.26	1.06 ± 0.23	0.94 ± 0.18	0.87 ± 0.18	0.80 ± 0.25	**0.59 ± 0.37**
estradiol	1.09 ± 0.16	1.10 ± 0.14	1.08 ± 0.09	1.09 ± 0.12	1.13 ± 0.24	1.22 ± 0.27	**1.35 ± 0.14**	**1.53 ± 0.17**	**1.77 ± 0.16**	**1.87 ± 0.21**

Mean ± SD. n=3. Statistically significant values are marked in bold.

### BPA

Following BPA exposure, concentrations of all measured androgens in supernatants were decreased in a dose-dependent matter at doses ≥10µM (**Figure 2A**): androstenedione (0.63 ± 0.05 fold-change in comparison to vehicle-treated controls, p ≤ 0.01 at 10µM; 0.37 ± 0.11, p ≤ 0.0001 at 25µM; 0.25 ± 0.10, p ≤ 0.0001 at 50µM; 0.20 ± 0.03, p ≤ 0.0001 at 100µM), testosterone (0.65 ± 0.05, p ≤ 0.01 at 10µM; 0.44 ± 0.14, p ≤ 0.0001 at 25µM; 0.37 ± 0.15, p ≤ 0.0001 at 50µM; 0.30 ± 0.03, p ≤ 0.0001), and dihydrotestosterone (0.47 ± 0.27, p ≤ 0.0001 at 25µM; 0.31 ± 0.10, p ≤ 0.0001; 0.19 ± 0.10, p ≤ 0.0001 at 100µM). Synthesis of DHEA (0.61 ± 0.14, p ≤ 0.05 at 25µM; 0.41 ± 0.27, p ≤ 0.001 at 50µM; 0.17 ± 0.10, p ≤ 0.0001 at 100µM) and DHEAS (0.76 ± 0.18, p ≤ 0.05 at 25µM; 0.41 ± 0.38, p ≤ 0.05 at 50µM; 0.33 ± 0.15, p ≤ 0.01) were also significantly lowered by BPA treatment.

Progesterone production (0.10 ± 0.10, p ≤ 0.0001) was only lowered at 100µM, while 17-hydroxyprogesterone was affected at >25µM (0.52 ± 0.21, p ≤ 0.05 at 25µM; 0.47 ± 0.36, p ≤ 0.001 at 50µM; 0.19 ± 0.05, p ≤ 0.0001 at 100µM). Additionally, their derivates 11-deoxycorticosterone (0.44 ± 0.10, p ≤ 0.001 at 25µM; 0.36 ± 0.14, p ≤ 0.001 at 50µM; 0.45 ± 0.22, p ≤ 0.001at 100µM), and 11-deoxycortisol (0.62 ± 0.08, p ≤ 0.0001 at 25µM; 0.46 ± 0.14, p ≤ 0.0001 at 50µM; 0.38 ± 0.08, p ≤ 0.0001 at 100µM) were decreased, respectively. However, secretion of estradiol was elevated at 100µM BPA (1.82 ± 0.47; p ≤ 0.001).

### BPF

BPF exposure caused a 2.04-, 4.04-, 4.58-, 6.32-fold-increase in 21-deoxycortisol levels at 1, 10, 25, and 50µM BPF respectively (**Figure 2B**). However, this was not statistically significant. At 100µM, it was further elevated to 16.88-fold (16.88 ± 4.01, p ≤ 0.05). Additionally, an increase of progesterone (2.39 ± 0.88 at 25µM; 2.85 ± 1.00 at 50µM, both not statistically significant; 5.13 ± 2.10; p ≤ 0.01 at 100µM), corticosterone (6.63 ± 2.75, p ≤ 0.05 at 100µM), 17-hydroxyprogesterone (2.57 ± 0.49, p ≤ 0.001 at 25µM; 2.65 ± 0.61, p ≤ 0.001 at 50µM; 2.55 ± 0.79; p ≤ 0.0001 at 100µM), and estradiol (1.72 ± 0.36, p ≤ 0.05 at 50µM; 3.60 ± 0.58; p ≤ 0.0001 at 100µM) was found after BPF treatment.

Concentration of cortisone (0.54 ± 0.08; p ≤ 0.01), 11-deoxycortisol (0.51 ± 0.10, p 0.05) DHEAS (0.46 ± 0.34; p ≤ 0.01), androstenedione (0.48 ± 0.08; p ≤ 0.05), and dihydrotestosterone (0.39 ± 0.20; p ≤ 0.001) decreased at 100µM BPF compared to control. Testosterone levels were lowered at >25µM (0.81 ± 0.14, p ≤ 0.05 at 25µM; 0.73 ± 0.05, p ≤ 0.01 at 50µM; 0.67 ± 0.20, p ≤ 0.001 at 100µM).

### BPS

After 72 hours of BPS exposure, a significant and dose-dependent decrease in the concentration of 11-deoxycorticosterone (0.56 ± 0.19, p ≤ 0.001 at 1µM; 0.24 ± 0.14, p ≤ 0.0001 at 10µM; 0.27 ± 0.18, p ≤ 0.0001 at 25µM; 0.22 ± 0.15, p ≤ 0.0001 at 50µM; 0.18 ± 0.09, p ≤ 0.0001 at 100µM), corticosterone (0.50 ± 0.11, p ≤ 0.0001 at 1µM; 0.26 ± 0.07, p ≤ 0.0001 at 10µM; 0.50 ± 0.14, p ≤ 0.0001 at 25µM; 0.40 ± 0.07, p ≤ 0.0001 at 50µM; 0.28 ± 0.07, p ≤ 0.0001 at 100µM), and 11-deoxycortisol (0.71 ± 0.16, p ≤ 0.001 at 1µM; 0.39 ± 0.13, p ≤ 0.0001 at 10µM; 0.37 ± 0.04, p ≤ 0.0001 at 25µM; 0.35 ± 0.06, p ≤ 0.0001 at 50µM; 0.25 ± 0.03, p ≤ 0.0001 at 100µM) was observed (**Figure 2C**).

While levels of progesterone (0.28 ± 0.09, p ≤ 0.05 at 100µM), aldosterone (0.70 ± 0.27, p ≤ 0.05 at 1µM; 0.54 ± 0.32, p ≤ 0.001 at 10µM; 0.71 ± 0.03, p ≤ 0.05 at 25µM; 0.61 ± 0.03, p ≤ 0.01 at 50µM; 0.50 ± 0.17, p ≤ 0.001 at 100µM), and cortisol (0.78 ± 0.13, p ≤ 0.05 at 1µM; 0.56 ± 0.09, p ≤ 0.01 at 10µM; 0.45 ± 0.10, p ≤ 0.0001 at 100µM) were found to be lowered compared to controls, 17-OH-progesterone (2.44 ± 0.61, p ≤ 0.01 at 10µM; 2.41 ± 0.47, p ≤ 0.01 at 25µM; 2.19 ± 0.20, p ≤ 0.05 at 50µM) and 21-deoxycortisol (2.25 ± 0.38 at 1µM; 3.34 ± 1.01 at 10µM, both not significant; 8.78 ± 1.00, p ≤ 0.0001 at 25µM; 6.81 ± 0.50, p ≤ 0.01 at 50µM; 2.01 ± 0.40, not significant) had a biphasic response with BPS treatment.

Similarly to BPA and BPF, all measured androgens were significantly suppressed: DHEA (0.57 ± 0.03, p ≤ 0.01 at 25µM; 0.55 ± 0.04, p ≤ 0.01 at 50µM; 0.48 ± 0.31, p ≤ 0.001 at 100µM), DHEAS (0.81 ± 0.14, p ≤ 0.05 at 1µM; 0.70 ± 0.14, p ≤ 0.001 at 10µM; 0.76 ± 0.02, p ≤ 0.01 at 25µM; 0.78 ± 0.04, p ≤ 0.01 at 50µM; 0.48 ± 0.01, p ≤ 0.0001 at 100µM), androstenedione (0.59 ± 0.42, p ≤ 0.01 at 100µM), testosterone (0.75 ± 0.02, p ≤ 0.05 at 25µM; 0.73 ± 0.07, p ≤ 0.05 at 50µM; 0.39 ± 0.18, p ≤ 0.0001 at 100µM), DHT (0.31 ± 0.06, p ≤ 0.0001 at 100µM). However, estradiol levels were again increased (1.40 ± 0.05, p ≤ 0.05 at 50µM; 1.36 ± 0.47, p ≤ 0.05 at 100µM).

### Bisphenol mixture

The combination of all three bisphenols led to a decreased concentration of 11-deoxycorticosterone (0.42 ± 0.05, p ≤ 0.001 at 10µM; 0.27 ± 0.04, p ≤ 0.0001 at 25µM; 0.23 ± 0.02, p ≤ 0.0001 at 50µM; 0.27 ± 0.08, p ≤ 0.0001 at 100µM), corticosterone (0.57 ± 0.32, p ≤ 0.05 at 25µM; 0.54 ± 0.30, p ≤ 0.01 at 50µM; 0.39 ± 0.22, p ≤ 0.001 at 100µM), and aldosterone (0.62 ± 0.06, p ≤ 0.01 at 100µM) (**Figure 2D**). Besides, levels of progesterone (0.63 ± 0.59, p ≤ 0.01 at 100µM), 11-deoxycortisol (0.61 ± 0.12, p ≤ 0.01 at 10µM; 0.45 ± 0.09, p ≤ 0.0001 at 25µM; 0.39 ± 0.06, p ≤ 0.0001 at 50µM; 0.36 ± 0.02, p ≤ 0.0001 at 100µM), and cortisol (0.76 ± 0.19, p ≤ 0.01 at 25µM; 0.70 ± 0.20, p ≤ 0.001 at 50µM; 0.53 ± 0.19, p ≤ 0.001 at 100µM) were lowered compared to control.

Meanwhile, levels of 17-OH-progesterone (1.83 ± 0.79, p ≤ 0.05 at 10µM), 21-deoxycortisol (2.92 ± 0.20, p ≤ 0,01 at 10µM; 3.21 ± 0.45, p ≤ 0.001 at 25µM; 2.92 ± 0.32, p ≤ 0.001 at 50µM), and estradiol (1.35 ± 0.14, p ≤ 0.05 at 10µM; 1.53 ± 0.17, p ≤ 0.001 at 25µM; 1.77 ± 0.16, p ≤ 0.0001 at 50µM; 1.87 ± 0.21, p ≤ 0.0001 at 100µM) were elevated, in a way comparable to exposure to BPF and BPS.

Regarding androgens, DHEAS (0.74 ± 0.18, p ≤ 0.05 at 25µM; 0.64 ± 0.23, p ≤ 0.01 at 50µM: 0.47 ± 0.29, p ≤ 0.0001 at 100µM), androstenedione (0.65 ± 0.22, p ≤ 0.01 at 50µM; 0.42 ± 0.18, p ≤ 0.0001 at 100µM), testosterone (0.70 ± 0.24, p ≤ 0.05 at 25µM; 0.59 ± 0.27, p ≤ 0.01 at 50µM; 0.47 ± 0.23, p ≤ 0.001 at 100µM), and DHT (0.59 ± 0.37, p ≤ 0.001 at 100µM) resulted in dose-dependent reductions after exposure to BPmix.

### Steroid ratio

To identify potentially affected metabolic-enzymes in steroidogenesis, the ratios between the product and the substrate of each CYP-dependent conversion were calculated for treated and untreated cells. The ratios of the treated cells were related to the vehicle-treated cells. Differences in these calculated ratios might indicate significant alterations in steroidogenesis after bisphenol exposure, whereas values >1 indicate an inhibition, and values<1 an enhancement of the respective steroidogenic step:


substrate productsubstrate (1%DMSO)product (1%DMSO)

### CYP11B1 and CYP11B2

Regarding CYP11B1(11β-hydroxylase)-mediated reactions, calculations revealed lowered ratios for all three observable steroidogenic steps (11-deoxyocorticosterone to corticosterone, 11-deoxycortisol to cortisol, and 17-hydroxyprogesterone to 21-deoxycortisol) following treatment with BPF (**Figures 3A–C**), BPS, and BPmix. For instance, BPF led to a reduced ratio of 11-deoxycortisol to cortisol at doses >10µM (0.71 ± 0.08-fold change compared to vehicle-treated controls, p ≤ 0.01 at 25µM; 0.64 ± 0.17, p ≤ 0.001 at 50µM; 0.53 ± 0.11, p ≤ 0.0001 at 100µM). These data indicate an almost 2-fold enhancement of 11β-hydroxylation in the steroidogenic pathway.

Aldosterone synthase activity, catalyzed by CYP11B2 seemed not to be affected by BPA, BPF, and BPS. However, the ratio of corticosterone to aldosterone was slightly reduced by BPmix (data not shown).

### CYP17A1

The two steps, which are mediated by CYP17A1, show different trends after bisphenol treatment: while the ratio of 17-hydroxyprogesterone to androstenedione, representing 17,20-lyase activity, is significantly enhanced by BPF (**Figure 3D**), BPS (**Figure 3E**), and BPmix (**Figure 3F**), the rate of progesterone to 17-hydroxyprogesteone conversion, representing 17α-hydroxylase activity, was not affected by BPA or BPF, and reduced by BPS (0.34 ± 0.03, p ≤ 0.05 at 10µM; 0.25 ± 0.05, p ≤ 0.05; 0.32 ± 0.17, p ≤ 0.05 at 50µM), BPmix (0.64 ± 0.01, p ≤ 0.01 at 10µM; 0.58 ± 0.05, p ≤ 0.01 at 25µM; 0.61 ± 0.05, p ≤ 0.01 at 50µM, both not shown). For example, the combinatory treatment led to a 2.26 ± 0.66-, and 2.47 ± 0.57-fold increased ratio of 17-hydroxyprogesterone to androstenedione at 25 and 50µM respectively. The ratio of progesterone to its hydroxylated derivate was reduced at doses >10µM to average (e.g. 0.64 ± 0.01, p ≤ 0.01 at 10µM; 0.59 ± 0.06, p ≤ 0.01 at 25µM) compared to controls. This however, indicates an inhibition of 17,20-lyase activity, assuming that this steroidogenic step is reduced to<50% compared to untreated cells. Meanwhile 17α-hydroxylase was either not affected or even enhanced by bisphenols.

### CYP19A1

Aromatase (CYP19A1), converting testosterone to estradiol seemed to be affected by all tested bisphenols and their mixture. The ratios were significantly reduced by BPA (**Figure 3G**), BPF (**Figure 3H**), BPS (**Figure 3I**), and BPmix (**Figure 3J**), indicating an enhancement of this step in the steroidogenic pathway. In a dose-dependent manner, CYP19-ratio was decreased by BPA at doses >10µM (0.51 ± 0.02 at 10µM; 0.38 ± 0.13 at 25µM; 0.25 ± 0.03 at 50µM; 0.18 ± 0.04 at 50µM, each with p ≤ 0.0001). Therefore, the conversion seemed to be enhanced by 2-fold (at 10µM) or even 4-fold (at 50µM).

### CYP21A2

The function of 21-hydroxylase (CYP21A2) was calculated for three reactions: progesterone to 11-deoxycorticosterone, 17-hydroxyprogesterone to 11-deoxycortisol, and 21-deoxycortisol to cortisol. CYP21A2-related ratios were conclusively increased by BPF (**Figure 3K**), BPS (**Figures 3L, M**), and BPmix (**Figure 3N**), indicating an inhibition of 21-hydroxylation in the presence of bisphenols. Significant increases in the calculated ratios were detected after BPS, leading to a >6-fold increase of 17-OHP to 11-deoxycortisol at doses >10µM (6.35 ± 0.82, p ≤ 0.01 at 10µM; 6.75 ± 1.70, p ≤ 0.01 at 25µM). Similarly dramatical increased are seen in the ratio of 21-deoxyocortisol to cortisol, with fold changes of 5.83 ± 0.79 at 10µM (p ≤ 0.01), 9.10 ± 2.99 at 25µM (p ≤ 0.0001), and 7.24 ± 1.64 at 50µM (p ≤ 0.001). No significant changes were induced by BPA.

### HSD3B2

HSD3B2-related ratio, calculated by DHEA’s conversion to androstenedione, was ambiguously affected by bisphenols. While the ratio was increased by BPA (1.65 ± 0.23, p ≤ 0.05 at 25µM; not shown) and BPmix (1.60 ± 0.21, p ≤ 0.01 at 50µM; 1.72 ± 0.29, p ≤ 0.001 at 100µM; **Figure 3O**), it was not affected by BPF, and even decrease by BPS treatment (0.70 ± 0.02, p ≤ 0.001 at 25µM; 0.69 ± 0.06, p ≤ 0.0001 at 50µM; not shown). This might indicate, that in the combinatory treatment, the effect of BPA on HSD3B2 was predominant.

### Others (HSD11B1/2, HSD17B3, SULT2A1, 5α-reductase)

Two other observable steps, mediated by hydroxysteroid-dehydrogenases were not conclusively influenced by bisphenol treatments: the ratio of cortisol to cortisone (HSD11B1/2) was slightly decreased by BPS and BPmix (0.76 ± 0.05, p ≤ 0.05 at 10µM; 0.70 ± 0.07, p ≤ 0.01 at 25µM; not shown), and the ratio of androstenedione to testosterone (HSD17B3), was only affected by BPA, in terms of a slight decrease (not shown).

Regarding sulfotransferase activity of SULT2A1 no trends were detected for the individual treatments with BPA, BPF, BPS. However, the ratio of DHEA to DHEAS was slightly increased by BPmix (0.69 ± 0.01, p ≤ 0.0001 at 50µM; not shown).

5α-reductase, expressed by the ratio of testosterone to dihydrotestosterone, was not affected by BPA, BPF, BPS or BPmix.

### Steroidogenic mRNA expression

mRNA expression of essential enzymes for adrenal steroidogenesis (StAR, CYP11B1, CYP11B2, CYP17A1, CYP19A1, CYP21A2, HSD3B2) were examined in the NCI-H295R cells after 72 hours exposure to vehicle or bisphenols. Fold-changes of the mRNA of these genes are presented in **Table 2**. The most relevant changes were seen for StAR, CYP11B1, and CYP17A1.

**Table 2:** mRNA expression of genes of interest: Fold change of treatment compared to vehicle-treated control ± SD.

Gene of interest		100nM	1µM	10µM	25µM	50µM
StAR	BPA	1.04 ± 0.18	1.27 ± 0.12	0.91 ± 0.16	1.07 ± 0.14	1.04 ± 0.07
	BPF	1.08 ± 0.25	1.10 ± 0.19	1.20 ± 0.21	1.03 ± 0.13	0.85 ± 0.21
	BPS	1.21 ± 0.57	0.98 ± 0.17	1.30 ± 0.45	**1.81 ± 0.25 (p=0.002)**	**2.15 ± 0.24** **(p<0.0001)**
	BPmix	1.14 ± 0.26	1.22 ± 0.32	**1.56 ± 0.53 (p=0.043)**	1.32 ± 0.30	1.02 ± 0.27
CYP11B1	BPA	1.01 ± 0.56	1.32 ± 0.75	**1.41** ± 0.21	0.51 ± 0.11	0.33 ± 0.15
	BPF	1.06 ± 0.32	**1.99 ± 0.51 (p=0.010)**	1.48 ± 0.33	**2.01 ± 0.71 (p=0.008)**	**1.80 ± 0.57 (p=0.048)**
	BPS	0.88 ± 0.22	0.75 ± 0,25	1.45 ± 0.60	0.40 ± 0.33	1.48 ± 0.50
	BPmix	1.36 ± 0.39	1.21 ± 0.21	0.65 ± 0.21	1.16 ± 0.16	0.94 ± 0.08
CYP11B2	BPA	0.90 ± 0.41	1.12 ± 0.29	0.68 ± 0.28	0.83 ± 0.12	0.48 ± 0.12
	BPF	1.55 ± 0.51	1.76 ± 0.78	1.88 ± 0.83	1.00 ± 0.75	1.35 ± 0.85
	BPS	1.25 ± 0.38	0.99 ± 0.33	1.12 ± 0.57	0.62 ± 0.20	0.85 ± 0.24
	BPmix	1.07 ± 0.50	0.87 ± 0.29	1.03 ± 0.32	1.14 ± 0.11	1.53 ± 0.42
CYP17A1	BPA	1.01 ± 0.18	1.09 ± 0.18	0.96 ± 0.08	1.03 ± 0.19	0.95 ± 0.15
	BPF	1.05 ± 0.18	**1.52 ± 0.35 (p=0.043)**	1.24 ± 0.28	1.26 ± 0.18	1.23 ± 0.30
	BPS	1.41 ± 0.53	1.55 ± 0.26	**1.84 ± 0.66 (p=0.005)**	**1.92 ± 0.27 (p=0.002)**	**1.95 ± 0.21 (p=0.001)**
	BPmix	1.06 ± 0.35	1.52 ± 0.36	1.11 ± 0.31	1.11 ± 0.44	1.07 ± 0.43
CYP19A1	BPA	1.34 ± 0.26	1.11 ± 0.31	0.98 ± 0.18	1.20 ± 0.29	1.12 ± 0.31
	BPF	0.59 ± 0.09	0.94 ± 0.29	0.80 ± 0.15	1.15 ± 0.15	1.35 ± 0.39
	BPS	1.12 ± 0.28	1.14 ± 0.30	0.90 ± 0.49	0.79 ± 0.20	1.03 ± 0.30
	BPmix	1.12 ± 0.53	0.84 ± 0.37	0.49 ± 0.42	1.10 ± 0.19	0.86 ± 0.16
CYP21A2	BPA	0.91 ± 0.29	1.27 ± 0.36	0.96 ± 0.37	1.44 ± 0.35	1.16 ± 0.37
	BPF	1.26 ± 0.16	1.01 ± 0.21	1.20 ± 0.70	0.64 ± 0.14	0.59 ± 0.16
	BPS	1.20 ± 0.23	1.34 ± 0.29	1.46 ± 0.64	0.98 ± 0.22	1.05 ± 0.24
	BPmix	1.10 ± 0.01	1.32 ± 0.80	0.91 ± 0.31	1.03 ± 0.54	0.83 ± 0.38
HSD3B2	BPA	0.81 ± 0.22	0.79 ± 0.47	0.81 ± 0.23	1.19 ± 0.50	0.95 ± 0.37
	BPF	0.91 ± 0.36	1.11 ± 0.63	1.34 ± 0.61	1.42 ± 0.70	1.15 ± 0.15
	BPS	0.82 ± 0.42	0.63 ± 0.24	1.04 ± 0.05	1.09 ± 0.10	1.16 ± 0.16
	BPmix	1.13 ± 0.14	0.89 ± 0.15	0.86 ± 0.21	1.25 ± 0.08	1.16 ± 0.60

n=3. Statistically significant results are printed in bold.

Treatment with BPS increased mRNA expression of StAR significantly at doses of 25 (1.81 ± 0.25-fold change compared to vehicle-treated controls, p ≤ 0.01) and 50µM (2.15 ± 0.24, p ≤ 0.0001), whereas 10µM of BPmix led to an increase by 1.56 ± 0.52 (p ≤ 0.05).

CYP11B1 expression was significantly affected by BPF at doses of 1µM (1.99 ± 0.51, p ≤ 0.01), 25µM (2.01 ± 0.71, p ≤ 0.01), and 50µM (1.80 ± 0.57, p ≤ 0.05).

Furthermore, altered levels of CYP17A1 mRNA were detected at BPF- and BPS-treated cells. BPF showed an effect at 1µM (1.52 ± 0.35, p ≤ 0.05), whereas BPS increased expression at 10µM (1.84 ± 0.66, p ≤ 0.01), 25µM (1.92 ± 0.27, p ≤ 0.01), and 50µM (1.95 ± 0.21, p ≤ 0.01).

## Discussion

In the present study, comprehensive steroidomics, including a set of 15 steroids in bisphenol-exposed adrenocortical cells, were analyzed for the first time, paying special attention to a combination of BPA and its substitutes BPF and BPS. LC-MS/MS measurements revealed dose-dependent and substance-specific alterations of steroid secretion at different non-cytotoxic concentrations. Of note, many steroids decreased following bisphenol treatment, but the fact that some steroids were significantly increased clearly indicates a specific effect of bisphenols on the adrenal gland beyond any non-specific cytotoxic cell suppression. Substrate-to-product ratio calculations revealed strongly altered activity of key steroidogenic enzymes, probably based on multiple molecular mechanisms.

The investigated bisphenols induced significant alterations of all analyzed steroid groups: progestogens (progesterone, 17-hydroxyprogesterone), mineralocorticoids (corticosterone, 11-deoxycorticosterone, aldosterone), glucocorticoids (11-deoxycortisol, 21-deoxycortisol, cortisol, cortisone), androgens (androstenedione, DHEA, DHEAS, testosterone, DHT), and estrogens (estradiol). The most pronounced effects were seen in relevant increases of estradiol after BPA exposure, while BPF and BPS treatment led to significantly increased levels of 21-deoxycortisol, (17-OH-) progesterone, corticosterone, and estradiol.

Reflecting “real life” conditions, where different contaminants can be detected in compounds, the combination of bisphenols A, F and S interfered with steroidogenesis resulting in elevated levels of 17-OH-progesterone, 21-deoxycortisol and estradiol. This combinatory treatment led to a steroidomic pattern as seen following BPF or BPS treatment only (see **Figure 4**), underlining that BPF and BPS, which are intended to replace BPA, may actually have a very relevant impact on adrenal steroidogenesis as well.

Additionally, all treatments led to significantly lowered androgens at non-cytotoxic doses, revealing that the known anti-androgenic activity of bisphenols extends to adrenal androgens, such as androstenedione and DHEA(S).

By calculating ratios of substrate and product of steroid metabolizing enzymes, dose-dependent alterations indicating an effect on the activity of these enzymes could be found. An inhibition of CYP17A1 associated 17,20-lyase activity and CYP21A2 can be certainly assumed, since substrate concentration outweighed the product concentration compared to control. Since these two crucial enzymes are dramatically inhibited, explaining the limited synthesis of effector hormones (e.g. cortisol), the accumulation of substrates (e.g. 17-hydroxyprogesterone), and a potential “backlog” effect favoring alternative pathways like the formation of 21-deoxycortisol.

The data further suggest an enhanced activity of CYP11B1- and CYP19-mediated steps, as product concentrations increased in relation to substrate concentrations following bisphenol treatment. Regarding aromatase activity, BPA was found to induce CYP19A1 gene and protein expression levels, and further increased conversion activity, promoting estradiol synthesis in testicular Leydig cells (11).

Meanwhile, HSD3B2-related ratios were only affected by the individual treatment with BPA and the combinatory BPmix. Interestingly, the ratios reveal alterations in conversion dynamics at lower doses than expected when considering results of isolated substances, with trends starting at 500nM already.

As Štefunková et al. reported significantly enhanced levels of intracellular cholesterol after treatment with BPA, BPF, and BPS, this might be explained by either increased cholesterol uptake or synthesis, or an accumulation due to inhibited mitochondrial uptake via StAR and CYP11A1, which serves as the first step in steroidogenesis (12). This might explain, why most of the measured steroids are decreased within higher doses of treatment. However, elevated doses of certain hormones, such as 17-hydroxyprogesterone or 21-deoxycortisol, and elevated expression of the StAR enzyme seem to contradict an overall shutdown of steroidogenesis by bisphenols.

In accordance with previous studies (12, 18) describing significantly increased concentrations of progesterone following BPF exposure, treatment with BPF resulted in 7.3-, 11.2-, and 12.7-fold increase at 30µM, 50µM, and 70µM BPF respectively. BPA led to lowered progesterone levels at >30µM (12, 18). However, in the current study, the effects (5.2-fold increase by 100µM BPF) were not as large as described before and, contrary to previous results, BPS did not alter progesterone significantly. Additionally, dramatically increased levels of 17-hydroxyprogesterone following BPF, BPS and BPmix exposure were seen. This might be of translational relevance, as administration of progestogenic contaminants during fetal development has led to virilization or feminization in animal models (23).

BPA and BPS exerted relevant anti-androgenic activity by significantly decreasing testosterone levels at doses >10µM. Additionally, BPF increased estradiol concentration at 100µM. Previous experiments detected similar results at low dose treatment with bisphenols, regarding lowered testosterone and increased estradiol (12, 24). The data support the hypothesis of anti-androgenic activity of bisphenols, as not only testosterone, but also essential adrenal androgens (DHEA, DHEAS, and androstenedione) were affected by single and combined exposure. As adrenal androgens act as precursor hormones for the formation of testosterone and are key players in the adrenarche (21), the presence of antiandrogenic endocrine disruptors could influence puberty timing and sexual maturation (22). Estradiol increased following treatment with all tested bisphenols, while calculation of CYP19A1 kinetics clearly suggests an increased conversion of testosterone to estradiol.

While endocrine disruptive effects on the reproductive system and sex steroid homeostasis have been frequently reported, the mechanistic impact on key adrenal function – the synthesis of gluco- and mineralocorticoids – is not conclusively understood. In our study, the most relevant glucocorticoid, cortisol was lowered by all tested bisphenols, while the mineralocorticoid aldosterone was affected only by BPA and BPS. This is in accordance with previous findings (18). While a decrease of most measured glucocorticoids (11-deoxycortisol, cortisol, and cortisone) could be detected, 21-deoxycortisol, which plays a minor physiological role, was strongly elevated by treatment with BPF, BPS and BPmix. This might be explained by an inhibited activity of 21-hydroxylase (CYP21A2), which preferably converts 17-hydroxyprogesterone to 11-deoxycortisol (25). However, its inhibition may promote the accumulation of substrates and alternative pathways. Therefore, 11β-hydroxylase activity (CYP11B1) and its expression could be enhanced by some bisphenols, leading to accumulation of 21-deoxycortisol. However, an anti-glucocorticoid activity was seen at relevant doses >10µM.Interestingly in the present experiments, treatment with the BPmix led to additive changes compared to the respective mono-treatments, as the individual concentration in the mix was only a third of the concentrations in the separate approaches. Although expected due to the similar structure of the tested bisphenols, this strongly suggests an additive disruptive effect of bisphenols and further supports the obvious assumption that the EDC problem must not be solved by substitution with structurally similar substances.

In contrast to previous studies Klicken oder tippen Sie hier, um Text einzugeben., significant changes in gene expression of essential steroidogenic enzymes, such as rate-limiting CYP11B2, CYP19A1, CYP21A2, or HSD3B2 were only inconclusively observed. As mRNA levels of StAR, CYP11B1, and CYP17A1 were enhanced compared to control, by BPF and BPS, effects on gene expression seem to contribute to the disruption of adrenal steroidogenesis.

Since gene expression analysis does not correlate with steroid alterations, other mechanism should be considered. Also, non-genetic modes of action, such as (post-)translational effects, allosteric modulation, interaction with redox partners or phosphorylation status of steroidogenic enzymes, or oxidative stress of the mitochondria and smooth endoplasmatic reticulum involved in steroidogenesis, could be relevant.

For instance, a CYP17A1-inhibiting activity of BPA has already been suggested, affecting 17, 20-lyase and 17a-hydroxylase on a functional level. Zhang et al. showed the inhibition of 17,20-lyase activity, while 17a-hydroxylase seemed not to be affected in H295R cells in the presence of BPA (26, 27). The present study supports this finding, as 17,20-lyase, converting 17-OH-progesterone to androstenedione, was significantly inhibited by BPF, BPS and BPmix, as respective ratios increased (see **Figures 3D–F**). Consistently, 17a-hydroxylase activity, representing the hydroxylation of progesterone, was either not relevantly altered by BPA, BPF (respective steroid ratios of around 1.0), or even enhanced by BPS and BPmix (ca. 2-fold induction). Meanwhile, enhanced CYP17A1 expression might be a compensatory cellular mechanism to ensure steroid production,

This dual effect suggests a mechanism that is not limited to gene/protein expression, as both functions of CYP17A1 would be affected in the same manner. As only the 17,20-lyase is dependent on P450 oxidoreductase and adequately phosphorylated serine and threonine residues (28), it is considerable that bisphenols interact with cytochrome b5 or the phosphorylation status of CYP17A1, thereby modulating its steroidogenic activity. As the activity of CYP21A2 is also highly dependent on its redox partner cytochrome b5 (29), and similar steroidomic trends are seen as with CYP17A1, this is consistent with bisphenols interfering with the steroidogenic pathways at enzymatic levels.

An accumulation of 17-hydroxyprogesterone and 21-deoxycortisol can be seen in congenital adrenal hyperplasia, caused by a hereditary dysfunction of 21-hydroxlase (CYP21A2) (30). As steroidomic patterns after treatment with BPF, BPS and BPmix revealed the same accumulations of precursors (e.g. progesterone) or alternative steroids (e.g. 21-deoxycortisol) and consequently lowered levels of mineralo- and glucocorticoid effector hormones, a specific blockade on CYP21A2-mediated steps seems to be obvious.

Furthermore, in silico analysis revealed the binding potential of BPA and its analogues to steroidogenic enzymes, which was comparable to CYP-inhibiting drugs, e.g. abiraterone. Most remarkable interactions were seen with CYP17A1, CYP21A2, and HSD3B2 (26). Thereby, allosteric modulation of steroidogenic enzymes by bisphenols is conceivable.

To draw a full picture, (31, 32) Bisphenols are known to promote the generation of radical oxygen species, and consecutive mitochondrial dysfunction, as well as lipid peroxidation in steroidogenic tissues (33, 34). Reduced mitochondrial activity was reported in the presence of bisphenols in H295R cells (12). If ROS-mediated mitochondrial dysfunction might explain the altered activity of mitochondrial CYP-enzymes, such as CYP11B1, remains speculative and should be part of future research.

As a further explanation, disrupted signaling pathways and (in-)direct effect on steroidogenesis might explain the presented results. Lan et al. showed BPA-induced phosphorylation of the JNK/c-Jun pathway affecting corticosterone secretion *in vivo* (19). Besides, the involvement of bisphenols in the disruption of ERK signaling (35) or the MAPK pathway (36) has been reported.

In total, this study, in accordance with previous results, indicates that bisphenols do not affect adrenal steroidogenesis via a single mechanism, but more likely mediate their disruptive effects in multiple molecular ways, including gene expression and the modulation of enzymatic activity,

### Limitations

It may be argued that concentrations of bisphenols above levels found in plasma or urine were used in this approach. On the other hand, it must be assumed that these substances accumulate in lipophilic tissue (such as the adrenal gland), especially following chronic exposure – considering that bisphenols are detectable in >92% of the general European population (9) therefore, higher levels in adrenal tissue than measurable in the blood is plausible. Nonetheless, *in vitro* studies are unable to reflect the hormonal feedback mechanisms including hypothalamus, pituitary gland, and autocrine processes. Furthermore, metabolites of bisphenols, formed *in vivo*, were not included in the analysis. In total, cell culture studies methodologically lack an easy transferability to real life conditions in terms of duration and levels of exposure, but nonetheless they are a valuable tool to detect detailed endocrine-disrupting effects in human cellular pathways.

### Conclusion

In conclusion, the extensive use and distribution of bisphenols over the last decades steers the attention to their potential influence on humans and the ecological system – especially as global resilience levels on the burden of synthetic chemicals are trespassed (37). This study presents in a comprehensive steroidomic approach strong *in-vitro* evidence that bisphenols alter adrenal steroidogenesis in multiple molecular ways and several target structures in the steroidogenic pathway could be identified (e.g. CYP11B1, CYP17A1, CYP21A2). BPF and BPS were shown to significantly disrupt adrenal steroidogenesis, Therefore, their use as substitute compounds for BPA seems rather unreasonable. In the future, further studies will address the impact of the shown impacts on living organism to evaluate the long-term consequences for wildlife and human health.

## Data availability statement

The original contributions presented in the study are included in the article/**Supplementary Material**, further inquiries can be directed to the corresponding author/s.

## Ethics statement

Ethical approval was not required for the studies on humans in accordance with the local legislation and institutional requirements because only commercially available established cell lines were used.

## Author contributions

BP: Writing – review & editing, Writing – original draft, Visualization, Methodology, Investigation, Data curation. LK: Writing – review & editing, Writing – original draft, Supervision, Methodology, Data curation, Conceptualization. SK: Writing – review & editing, Resources, Data curation. HS: Writing – review & editing, Supervision. MK: Writing – review & editing, Supervision, Resources, Methodology. MF: Writing – review & editing, Supervision, Resources, Project administration, Funding acquisition. UD: Writing – review & editing, Supervision, Resources, Project administration, Funding acquisition, Conceptualization.
