# Supplementary material for: Disruptive effects of plasticizers bisphenol A, F, and S on steroidogenesis of adrenocortical cells
Source: Front Endocrinol (Lausanne). 2024 Jun 20;15:1387133. doi: 10.3389/fendo.2024.1387133 (PMC11222671; doi:10.3389/fendo.2024.1387133)
Supplement: Supplementary file 1 [file DataSheet_1.docx]

Supplementary Tab 1.: Overview of used primer sequences

| Gene of interest | Hs |
| --- | --- |
| StAR | Hs00986559_g1 |
| CYP11B1 | Hs00357016_g1 |
| CYP11B2 | Hs01597732_m1 |
| CYP17A1 | Hs01124136_m1 |
| CYP19A1 | Hs00903411_m1 |
| CYP21A2 | Hs00365736_g1 |
| HSD3B2 | Hs00605123_m1 |
| ACTB | Hs01060665_g1 |

Supplementary Tab. 2: Substrate-to-product ratios of fifteen steroidogenic conversions, calculated from measured steroid levels after 72h of treatment with BPA, BPF, BPS, or BPmix. Mean±SD. n=3.

| **BPA** |  | 1nM | 50nM | 100nM | 250nM | 500nM | 1µM | 10µM | 25µM | 50µM | 100µM |
| --- | --- | --- | --- | --- | --- | --- | --- | --- | --- | --- | --- |
| **CYP11B1** | 17-OH-progesterone/21-deoxycortisol | 1.10±0.04 | 1.14±0.11 | 1.06±0.05 | 0.88±0.06 | 1.10±0.10 | 1.11±0.12 | 0.87±0.09 | 1.02±0.12 | 0.91±0.50 | 0.42±0.72 |
| **CYP11B1** | 11-deoxycortisol/cortisol | 1.01±0.04 | 1.02±0.06 | 1.01±0.07 | 1.02±0.10 | 0.90±0.24 | 1.05±0.19 | 0.88±0.13 | 0.93±0.12 | 0.95±0.47 | 1.04±0.07 |
| **CYP11B1** | 11-deoxycorticosterone/corticosterone | 1.01±0.16 | 0.98±0.09 | 0.99±0.12 | 0.89±0.21 | 0.90±0.18 | 0.91±0.13 | 0.62±0.09 | 0.56±0.05 | 0.56±0.13 | 0.80±0.55 |
| **CYP11B2** | corticosterone/aldosterone | 1.09±0.09 | 1.22±0.55 | 1.07±0.15 | 1.25±0.34 | 0.98±0.16 | 0.92±0.08 | 1.19±0.16 | 1.30±0.25 | 1.21±0.42 | 0.85±0.27 |
| **CYP17A1** | progesterone/17-OH-progesterone | 1.10±0.33 | 1.08±0.19 | 1.13±0.25 | 1.10±0.44 | 1.15±0.30 | 1.15±0.32 | 1.00±0.18 | 0.89±0.07 | 0.89±0.11 | 0.65±0.27 |
| **CYP17A1** | 17-OH-progesterone/androstenedione | 1.06±0.21 | 1.14±0.16 | 1.44±0.43 | 1.53±0.85 | 1.53±0.65 | 0.79±0.69 | 1.07±0.93 | 1.64±0.09 | 1.72±0.59 | 0.59±0.52 |
| **CYP19A1** | testosterone/estradiol | 0.97±0.06 | 0.96±0.04 | 0.93±0.03 | 0.96±0.02 | 0.94±0.05 | 0.89±0.06 | 0.51±0.02 | 0.38±0.13 | 0.24±0.03 | 0.18±0.04 |
| **CYP21A2** | progesterone/11-deoxycorticosterone | 1.18±0.23 | 1.11±0.16 | 1.17±0.18 | 1.07±0.27 | 1.15±0.20 | 1.30±0.23 | 1.22±0.25 | 1.23±0.12 | 1.12±0.57 | 0.32±0.15 |
| **CYP21A2** | 17-OH-progesterone/11-deoxycortisol | 1.06±0.08 | 1.02±0.03 | 1.08±0.03 | 1.01±0.09 | 1.05±0.12 | 1.09±0.13 | 1.09±0.06 | 1.09±0.22 | 0.94±0.43 | 0.53±0.19 |
| **CYP21A2** | 21-deoxycortisol/cortisol | 0.98±0.03 | 0.91±0.06 | 1.03±0.06 | 1.17±0.11 | 0.88±0.34 | 1.03±0.17 | 1.10±0.10 | 0.99±0.19 | 1.06±0.61 | 0.22±0.38 |
| **HSD3B2** | DHEA/androstenedione | 1.02±0.08 | 1.03±0.09 | 1.02±0.06 | 1.02±0.19 | 1.03±0.05 | 1.03±0.09 | 1.49±0.14 | 1.65±0.23 | 1.53±0.37 | 0.91±0.57 |
| **HSD11B1/2** | cortisol/cortisone | 0.94±0.08 | 0.97±0.07 | 0.97±0.06 | 1.05±0.05 | 1.02±0.08 | 0.86±0.12 | 1.02±0.03 | 0.75±0.08 | 0.65±0.25 | 0.77±0.11 |
| **HSD17B3** | androstenedione/testosterone | 0.96±0.07 | 0.93±0.07 | 0.97±0.09 | 1.00±0.11 | 0.92±0.08 | 0.93±0.02 | 0.96±0.04 | 0.86±0.03 | 0.69±0.00 | 0.62±0.07 |
| **SULT2A1** | DHEA/DHEAS | 0.97±0.08 | 1.00±0.12 | 1.03±0.13 | 1.04±0.21 | 0.93±0.12 | 0.91±0.04 | 1.24±0.17 | 1.14±0.20 | 0.88±0.09 | 0.43±0.37 |
| **5α-reductase** | testosterone/DHT | 0.90±0.10 | 0.90±0.12 | 0.87±0.06 | 0.96±0.09 | 1.01±0.14 | 0.88±0.09 | 0.82±0.10 | 1.00±0.21 | 1.17±0.09 | 2.26±1.76 |

| **BPF** |  | 1nM | 50nM | 100nM | 250nM | 500nM | 1µM | 10µM | 25µM | 50µM | 100µM |
| --- | --- | --- | --- | --- | --- | --- | --- | --- | --- | --- | --- |
| **CYP11B1** | 17-OH-progesterone/21-deoxycortisol | 0.81±0.15 | 0.92±0.14 | 0.85±0.07 | 0.92±0.16 | 0.72±0.12 | 0.59±0.03 | 0.44±0.07 | 0.60±0.11 | 0.52±0.17 | 0.33±0.19 |
| **CYP11B1** | 11-deoxycortisol/cortisol | 1.00±0.01 | 1.03±0.02 | 1.02±0.04 | 1.03±0.04 | 0.93±0.04 | 0.87±0.02 | 0.87±0.05 | 0.71±0.08 | 0.64±0.17 | 0.53±0.11 |
| **CYP11B1** | 11-deoxycorticosterone/corticosterone | 0.92±0.20 | 0.94±0.11 | 0.84±0.05 | 0.87±0.11 | 0.69±0.03 | 0.67±0.05 | 0.50±0.07 | 0.45±0.01 | 0.41±0.06 | 0.23±0.14 |
| **CYP11B2** | corticosterone/aldosterone | 1.02±0.09 | 1.00±0.11 | 1.09±0.01 | 1.15±0.15 | 1.10±0.09 | 1.03±0.17 | 1.19±0.19 | 1.16±0.17 | 1.17±0.20 | 3.29±1.48 |
| **CYP17A1** | progesterone/17-OH-progesterone | 1.11±0.18 | 1.10±0.22 | 0.97±0.15 | 1.05±0.13 | 0.82±0.13 | 0.84±0.16 | 0.69±0.08 | 0.91±0.12 | 1.03±0.25 | 1.93±0.52 |
| **CYP17A1** | 17-OH-progesterone/androstenedione | 0.89±0.20 | 0.88±0.16 | 0.88±0.05 | 0.87±0.17 | 0.81±0.09 | 0.96±0.11 | 1.37±0.25 | 3.31±0.42 | 3.83±1.01 | 5.38±1.51 |
| **CYP19A1** | testosterone/estradiol | 1.03±0.10 | 1.03±0.09 | 1.05±0.03 | 1.07±0.07 | 1.13±0.05 | 1.06±0.06 | 0.88±0.07 | 0.51±0.02 | 0.43±0.05 | 0.21±0.07 |
| **CYP21A2** | progesterone/11-deoxycorticosterone | 1.00±0.08 | 1.03±0.12 | 1.04±0.14 | 0.98±0.04 | 0.90±0.14 | 1.04±0.28 | 1.38±0.32 | 3.26±0.91 | 4.35±2.38 | 6.21±3.20 |
| **CYP21A2** | 17-OH-progesterone/11-deoxycortisol | 0.89±0.15 | 0.90±0.13 | 0.96±0.02 | 0.93±0.14 | 0.86±0.10 | 0.90±0.01 | 1.42±0.25 | 3.69±0.64 | 4.58±1.67 | 4.89±0.52 |
| **CYP21A2** | 21-deoxycortisol/cortisol | 1.11±0.08 | 1.01±0.08 | 1.16±0.10 | 1.05±0.07 | 1.12±0.06 | 1.34±0.08 | 2.90±0.80 | 4.45±1.08 | 5.94±2.72 | 12.56±8.81 |
| **HSD3B2** | DHEA/androstenedione | 0.97±0.09 | 1.05±0.06 | 1.02±0.08 | 0.96±0.12 | 0.99±0.06 | 1.03±0.03 | 1.01±0.11 | 0.97±0.05 | 1.01±0.02 | 1.35±0.17 |
| **HSD11B1/2** | cortisol/cortisone | 1.18±0.24 | 1.09±0.16 | 1.10±0.06 | 1.16±0.13 | 1.18±0.13 | 1.15±0.07 | 1.26±0.13 | 1.09±0.15 | 1.17±0.24 | 2.07±1.01 |
| **HSD17B3** | androstenedione/testosterone | 1.01±0.07 | 1.03±0.05 | 1.06±0.04 | 1.09±0.06 | 1.02±0.15 | 0.93±0.11 | 0.94±0.06 | 0.98±0.10 | 0.97±0.11 | 0.72±0.07 |
| **SULT2A1** | DHEA/DHEAS | 1.07±0.15 | 1.13±0.11 | 1.14±0.11 | 1.13±0.15 | 1.07±0.09 | 0.96±0.04 | 1.23±0.16 | 0.98±0.07 | 0.99±0.07 | 1.67±0.65 |
| **5α-reductase** | testosterone/DHT | 1.19±0.16 | 1.16±0.13 | 1.14±0.11 | 1.20±0.13 | 1.31±0.03 | 1.18±0.03 | 1.19±0.11 | 1.03±0.19 | 1.03±0.16 | 2.49±0.81 |

| **BPS** |  | 1nM | 50nM | 100nM | 250nM | 500nM | 1µM | 10µM | 25µM | 50µM | 100µM |
| --- | --- | --- | --- | --- | --- | --- | --- | --- | --- | --- | --- |
| **CYP11B1** | 17-OH-progesterone/21-deoxycortisol | 0.86±0.07 | 0.91±0.17 | 0.94±0.10 | 1.00±0.09 | 0.79±0.16 | 0.81±0.14 | 0.74±0.05 | 0.29±0.05 | 0.33±0.04 | 0.78±0.06 |
| **CYP11B1** | 11-deoxycortisol/cortisol | 0.99±0.04 | 0.99±0.06 | 1.01±0.05 | 1.03±0.04 | 0.98±0.04 | 0.90±0.04 | 0.69±0.11 | 0.39±0.05 | 0.37±0.05 | 0.57±0.10 |
| **CYP11B1** | 11-deoxycorticosterone/corticosterone | 0.97±0.12 | 0.98±0.09 | 0.99±0.10 | 1.07±0.07 | 0.97±0.10 | 1.10±0.12 | 0.88±0.31 | 0.48±0.21 | 0.51±0.26 | 0.61±0.16 |
| **CYP11B2** | corticosterone/aldosterone | 0.81±0.24 | 0.90±0.29 | 0.92±0.27 | 0.81±0.24 | 0.75±0.23 | 0.53±0.19 | 0.34±0.13 | 0.71±0.15 | 0.64±0.07 | 0.42±0.09 |
| **CYP17A1** | progesterone/17-OH-progesterone | 0.65±0.22 | 0.82±0.33 | 0.74±0.27 | 0.78±0.29 | 0.63±0.25 | 0.60±0.25 | 0.37±0.15 | 0.34±0.03 | 0.25±0.05 | 0.32±0.17 |
| **CYP17A1** | 17-OH-progesterone/androstenedione | 1.03±0.03 | 1.01±0.09 | 1.04±0.04 | 1.21±0.18 | 1.22±0.30 | 1.83±0.37 | 2.57±0.55 | 2.98±0.51 | 2.77±0.30 | 2.59±0.68 |
| **CYP19A1** | testosterone/estradiol | 1.04±0.01 | 0.99±0.04 | 1.02±0.04 | 0.97±0.01 | 1.03±0.06 | 0.97±0.02 | 0.57±0.03 | 0.53±0.05 | 0.79±0.09 | 0.29±0.03 |
| **CYP21A2** | progesterone/11-deoxycorticosterone | 0.86±0.32 | 0.89±0.37 | 0.87±0.35 | 1.19±0.55 | 1.30±0.68 | 2.33±1.26 | 4.88±2.85 | 6.24±5.75 | 4.49±3.61 | 1.94±0.51 |
| **CYP21A2** | 17-OH-progesterone/11-deoxycortisol | 1.13±0.02 | 1.05±0.08 | 1.12±0.09 | 1.40±0.16 | 1.54±0.33 | 2.61±0.60 | 6.35±0.82 | 6.75±1.70 | 6.42±1.56 | 6.21±4.07 |
| **CYP21A2** | 21-deoxycortisol/cortisol | 1.30±0.06 | 1.17±0.09 | 1.21±0.03 | 1.44±0.09 | 1.91±0.16 | 2.88±0.19 | 5.83±0.79 | 9.10±2.98 | 7.24±1.64 | 4.11±1.98 |
| **HSD3B2** | DHEA/androstenedione | 0.95±0.05 | 0.98±0.04 | 0.96±0.05 | 0.98±0.04 | 0.97±0.05 | 0.94±0.08 | 0.85±0.02 | 0.70±0.02 | 0.69±0.06 | 0.84±0.06 |
| **HSD11B1/2** | cortisol/cortisone | 0.99±0.05 | 1.02±0.07 | 1.01±0.08 | 0.91±0.08 | 0.95±0.12 | 0.78±0.09 | 0.56±0.07 | 0.76±0.24 | 0.62±0.18 | 0.60±0.07 |
| **HSD17B3** | androstenedione/testosterone | 1.02±0.03 | 1.04±0.03 | 1.04±0.02 | 1.07±0.05 | 1.09±0.03 | 1.05±0.02 | 1.17±0.05 | 1.08±0.01 | 1.08±0.04 | 1.37±0.30 |
| **SULT2A1** | DHEA/DHEAS | 1.01±0.02 | 1.05±0.08 | 1.05±0.01 | 1.12±0.09 | 1.19±0.19 | 1.15±0.18 | 1.19±0.22 | 0.75±0.03 | 0.70±0.06 | 1.23±0.49 |
| **5α-reductase** | testosterone/DHT | 1.07±0.03 | 1.02±0.06 | 1.04±0.07 | 1.01±0.05 | 1.12±0.12 | 0.98±1.10 | 0.88±0.13 | 0.83±0.04 | 0.77±0.07 | 1.28±0.33 |

| **BPmix** |  | 1nM | 50nM | 100nM | 250nM | 500nM | 1µM | 10µM | 25µM | 50µM | 100µM |
| --- | --- | --- | --- | --- | --- | --- | --- | --- | --- | --- | --- |
| **CYP11B1** | 17-OH-progesterone/21-deoxycortisol | 1.05±0.11 | 1.05±0.07 | 1.00±0.07 | 0.99±0.10 | 0.98±0.10 | 0.90±0.11 | 0.72±0.15 | 0.68±0.15 | 0.71±0.19 | 0.98±0.29 |
| **CYP11B1** | 11-deoxycortisol/cortisol | 0.98±0.03 | 0.99±0.02 | 0.98±0.01 | 0.99±0.01 | 0.97±0.03 | 0.93±0.02 | 0.73±0.09 | 0.61±0.10 | 0.57±0.06 | 0.73±0.16 |
| **CYP11B1** | 11-deoxycorticosterone/corticosterone | 1.09±0.11 | 1.04±0.12 | 1.02±0.05 | 0.66±0.46 | 0.67±0.48 | 0.70±0.51 | 0.52±0.44 | 0.41±0.35 | 0.36±0.30 | 0.49±0.45 |
| **CYP11B2** | corticosterone/aldosterone | 0.97±0.01 | 0.98±0.08 | 1.01±0.02 | 0.70±0.50 | 0.62±0.44 | 0.59±0.42 | 0.48±0.35 | 0.48±0.36 | 0.46±0.35 | 0.42±0.33 |
| **CYP17A1** | progesterone/17-OH-progesterone | 1.03±0.03 | 1.02±0.05 | 1.00±0.13 | 1.00±0.16 | 0.95±0.13 | 0.87±0.12 | 0.64±0.02 | 0.58±0.06 | 0.61±0.05 | 0.59±0.09 |
| **CYP17A1** | 17-OH-progesterone/androstenedione | 0.97±0.04 | 0.97±0.05 | 0.98±0.10 | 0.99±0.14 | 0.96±0.04 | 1.09±0.12 | 1.87±0.47 | 2.26±0.66 | 2.57±0.57 | 2.11±0.57 |
| **CYP19A1** | testosterone/estradiol | 0.96±0.09 | 0.94±0.10 | 0.94±0.09 | 0.91±0.09 | 0.93±0.13 | 0.86±0.10 | 0.62±0.11 | 0.46±0.12 | 0.33±0.12 | 0.25±0.12 |
| **CYP21A2** | progesterone/11-deoxycorticosterone | 1.03±0.02 | 1.04±0.04 | 1.03±0.16 | 1.10±0.25 | 1.13±0.10 | 1.27±0.28 | 2.76±0.78 | 4.04±1.57 | 4.16±1.46 | 3.94±2.31 |
| **CYP21A2** | 17-OH-progesterone/11-deoxycortisol | 1.01±0.04 | 0.99±0.06 | 1.02±0.10 | 1.06±0.15 | 1.12±0.12 | 1.30±0.19 | 2.92±0.66 | 4.06±1.36 | 4.25±1.63 | 2.69±1.80 |
| **CYP21A2** | 21-deoxycortisol/cortisol | 0.94±0.11 | 0.94±0.11 | 1.01±0.12 | 1.06±0.13 | 1.13±0.20 | 1.37±0.25 | 3.15±1.12 | 3.88±1.84 | 3.81±2.19 | 2.25±1.79 |
| **HSD3B2** | DHEA/androstenedione | 1.02±0.02 | 1.02±0.01 | 1.01±0.09 | 1.02±0.08 | 1.05±0.09 | 1.03±0.11 | 1.09±0.05 | 1.32±0.16 | 1.60±0.21 | 1.72±0.29 |
| **HSD11B1/2** | cortisol/cortisone | 0.99±0.01 | 1.01±0.04 | 1.04±0.06 | 1.01±0.09 | 1.04±0.06 | 0.99±0.06 | 0.76±0.05 | 0.70±0.07 | 0.70±0.09 | 0.69±0.18 |
| **HSD17B3** | androstenedione/testosterone | 1.08±0.12 | 1.08±0.11 | 1.11±0.12 | 1.13±0.12 | 1.16±0.11 | 1.15±0.13 | 1.18±0.18 | 1.18±0.19 | 1.14±0.15 | 0.93±0.08 |
| **SULT2A1** | DHEA/DHEAS | 1.06±0.03 | 1.08±0.02 | 1.10±0.07 | 1.14±0.08 | 1.22±0.08 | 1.20±0.08 | 1.26±0.17 | 1.45±0.22 | 1.66±0.25 | 1.61±0.17 |
| **5α-reductase** | testosterone/DHT | 0.94±0.08 | 0.95±0.06 | 0.95±0.04 | 0.93±0.05 | 0.97±0.12 | 0.98±0.12 | 0.89±0.12 | 0.79±0.08 | 0.74±0.10 | 0.86±0.24 |
